# γ-Glutamyl transpeptidase-activatable near-infrared nanoassembly for tumor fluorescence imaging-guided photothermal therapy

**DOI:** 10.7150/thno.60586

**Published:** 2021-05-13

**Authors:** Fangyuan Zhou, Shikui Yang, Chao Zhao, Wangwang Liu, Xufeng Yao, Hui Yu, Xiaolian Sun, Yi Liu

**Affiliations:** 1 School of Engineering, China Pharmaceutical University, Nanjing 211198, P. R. China.; 2 State Key Laboratory of Natural Medicines, Key Laboratory of Drug Quality Control and Pharmacovigilance, Department of Pharmaceutical Analysis, China Pharmaceutical University, Nanjing 211198, P. R. China.

**Keywords:** γ-glutamyl transpeptidase, near-infrared probe, self-assembly, photothermal therapy, tumor microenvironment

## Abstract

**Rationale:** Precise treatment of tumors is attracting increasing attention. Molecular probes simultaneously demonstrating the diagnostic signal and pharmacological effect in response to tumor microenvironment are highly desired. γ-glutamyl transpeptidase (GGT) is a biomarker with significantly up-regulated expression in the tumor area. We developed a GGT responsive near-infrared (NIR) nanoassembly for tumor-specific fluorescence imaging-guided photothermal therapy.

**Methods:** The GGT responsive NIR probe was constructed by conjugating GGT-specific substrate γ-glutamic acid (γ-Glu) with cyanine fluorophore (NRh-NH_2_) via amide reaction. The resulting NRh-G spontaneously assembled into nanoparticles (NRh-G-NPs) around 50 nm. The NPs were characterized and the properties evaluated in the presence or absence of GGT. Subsequently, we studied fluorescence imaging and photothermal therapy of NRh-G-NPs *in vitro* and *in vivo*.

**Results:** NRh-G-NPs, upon specific reaction with GGT, turned into NRh-NH_2_-NPs, showing a ~180-fold fluorescence enhancement and excellent photothermal effect recovery. NRh-G-NPs could selectively light up U87MG tumor cells while their fluorescence was weak in L02 human normal liver cells. The NPs also showed excellent tumor cell ablation upon laser irradiation. After intravenous injection into tumor-bearing mice, NRh-G-NPs could arrive in the tumor area and specifically light up the tumor. Following laser irradiation, the tumor could be completely erased with no tumor reoccurrence for up to 40 days.

**Conclusions:** NRh-G-NPs were specifically responsive to GGT overexpressed in U87MG tumor cells and selectively lit up the tumor for imaging-guided therapy. Besides, the recovery of photothermal property in the tumor area could improve cancer therapy precision and decreased side effects in normal tissues.

## Introduction

Cancer poses a significant threat to human health and life 1-3. Besides various cancer therapeutic methods, such as surgery, radiotherapy, and chemotherapy, photothermal therapy (PTT) has recently attracted much attention due to its high effectiveness and specificity 4-8. With the help of photothermal conversion agents, the light irradiation can be absorbed and transferred into heat, destroying cancer tissues with minimal side effects 9, 10. The ideal photothermal conversion agents should be non-toxic, with high tumor-targeting and efficient photothermal conversion capabilities 11-14. Furthermore, the self-imaging capability is preferred to identify the accumulation of photothermal agents to better guide the laser operation timing and dosage 15-18.

Among a variety of inorganic and organic photothermal agents, organic dyes are preferred because of their fast excretion, excellent biocompatibility, and high clinical translation ability 19. Organic dyes based on cyanine and diketopyrrolopyrrole have higher penetration depth under the excitation of near-infrared (NIR) light, and therefore represent hot research areas in the tumor theranostics field 20. Nano-delivery systems, such as liposomes and nano-assemblies, have been used to improve solubility and tumor accumulation. Recently, smart nano-systems, whose diagnostic and therapeutic functions could be selectively activated in tumor area by either the tumor microenvironment (e.g. pH, hypoxia, enzyme) or external stimuli (e.g. light, ultrasound, magnetism), have emerged that have higher theranostic selectivity and efficiency 21-25.

Here, we have synthesized a γ-glutamyl transpeptidase (GGT)-responsive NIR cyanine fluorophore (NRh-G) by conjugating GGT-specific substrate γ-glutamic acid (γ-Glu) with cyanine fluorophore NRh-NH_2_ via amide reaction 26. The NRh-G spontaneously assembled into 50-60 nm nanospheres NRh-G-NPs in water and maintained the OFF state in the normal physiological environment. In the tumor cells overexpressing GGT, γ-Glu moieties on NRh-G-NPs were specifically recognized and cut off to form NRh-NH_2_-NPs, turning on the fluorescence and photothermal capabilities 27, thus maximizing the treatment specificity and accuracy 28.

## Methods

### Materials

Hyperpure water was used to prepare all aqueous solutions. Most materials were purchased from Shanghai Aladdin Bio-Chem Technology Co., Ltd. Boc-Glu-OtBu was purchased from Bidepharm. GGT was purchased from Wako Pure Chemical Industries, Ltd. Azaserine was purchased from Cayman. Sodium hippurate was purchased from Aladdin. If not noted, all the chemicals used in the experiments were of analytical reagent grade and used without further purification.

### Synthesis of NRh-G

NRh-NH_2_ was synthesized according to the method reported in the literature 29. Please refer to the supporting information for detailed synthesis steps (**Figure S1**).

NRh-NH_2_ (0.47 g, 1.0 mmol) was dissolved in dry dichloromethane (2.0 mL). 2-(7-Azabenzotriazol-1-yl)-N, N, N', N'-tetramethyluronium hexafluorophosphate (1.40 g, 5.0 mmol), Boc-Glu-OtBu (3.03 g, 10.0 mmol) and N, N-Diisopropylethylamine (20.0 μL) were dissolved in dry dichloromethane (20.0 mL) at 20 °C for 40 min. Then the former was dripped into the latter. The mixture was stirred at room temperature overnight under the nitrogen atmosphere. After the reaction, an appropriate amount of anhydrous magnesium sulfate was added to the solution to remove the water generated by the reaction. After filtration, the solvent was removed under vacuum filtration at 35 °C. The crude product was purified by silica column chromatography using CH_2_Cl_2_/CH_3_OH (v/v, 50/1) as the eluent to produce the NRh-G-Boc compound as the dark blue solid (0.38 g, yield 80%). NRh-G-Boc (0.73 g, 1.0 mmol), trifluoroacetic acid (20.0 μL) were placed in a flask containing dry dichloromethane (2.0 mL). The mixture was stirred at room temperature overnight. The solvent was removed under vacuum filtration at 35 °C. The crude product was purified by reversed phase C18 column chromatography using CH_3_OH /H_2_O (v/v, 4/1) as the eluent to produce the NRh-G compound as a dark blue solid (0.36 g, yield 50%). The structures were determined using mass spectrum (MS) (**Figure S2-S4**), high resolution mass spectrometry (HRMS) (**Figure S5**), ^1^H nuclear magnetic resonance (NMR) (**Figure S6-S9**), ^13^C NMR (**Figure S10**), and high performance liquid chromatography (HPLC) (**Figure S11**). ^1^H NMR (400 MHz, MeOD) δ 8.85 (t, *J* = 12.0 Hz, 1H), 8.36 (t, *J* = 9.5 Hz, 1H), 8.16 (d, *J* = 8.9 Hz, 1H), 8.09 (d, *J* = 1.8 Hz, 2H), 7.82 (d, *J* = 8.9 Hz, 1H), 7.78-7.72 (m, 1H), 7.66-7.60 (m, 1H), 7.44 (t, *J* = 9.1 Hz, 1H), 7.37-7.31 (m, 2H), 6.60 (dd, *J* = 16.0, 8.2 Hz, 1H), 4.60-4.51 (m, 2H), 3.76-3.70 (m, 1H), 2.82-2.79 (m, 2H), 2.74 (dd, *J* = 13.3, 6.7 Hz, 4H), 2.30-2.25 (m, 2H), 2.10 (s, 6H), 2.00-1.94 (m, 2H), 1.57 (d, *J* = 7.3 Hz, 3H). ^13^C NMR (75 MHz, DMSO) δ 175.46, 162.38, 155.91, 154.00, 141.92, 139.67, 139.14, 137.80, 134.36, 132.15, 131.09, 130.55, 129.71, 129.03, 128.38, 128.11, 127.70, 125.71, 123.35, 122.55, 114.22, 113.34, 112.23, 100.43, 96.36, 55.42, 51.46, 46.83, 27.85, 27.18, 24.10, 20.67, 13.00. MS (ESI^+^): calculated for C_36_H_38_N_3_O_4_^+^, 576.3 [M]^+^; found, 576.4 [M]^+^. HRMS (ESI^+^): calculated for C_36_H_38_N_3_O_4_^+^, 576.28568 [M]^+^; found, 576.28571 [M]^+^.

### Formation, characterization, and detection of NRh-G-NPs

The DMSO solution of NRh-G (1.0 mM) was dripped into water. Next, the mixture was stirred for 2 h 30-33 and DMSO was then removed by dialysis. As synthesized nanoparticles were characterized by transmission electron microscope (TEM), dynamic light scattering (DLS), and Zeta potential analyzer. The hydrodynamic size of nanoparticles was observed from a DLS study 34.

### Reaction of NRh-G-NPs with GGT in solution

NRh-G-NPs (5.8 μg/mL) were incubated with GGT (0.4 U/L) at 37 °C for 30 min. The UV absorption spectra of the solution before and after reaction from 400 to 850 nm were recorded by ultraviolet spectrophotometer. NRh-G-NPs (5.8 μg/mL) were incubated with GGT (0.4 U/L) at 37 °C for varying amounts of time. Next NRh-G-NPs (5.8 μg/mL) were incubated with different concentrations of GGT (from 0 U/L to 1.0 U/L) at 37 °C for 30 min. The fluorescence spectra from 700 to 900 nm were recorded by fluorescence synchronous scanning (excitation, 658 nm; emission, 740 nm). The selectivity of NRh-G-NPs (5.8 μg/mL) toward GGT was next examined by incubating it with GGT, KCl, NaCl, MgCl_2_, CaCl_2_, Cysteine, glutathione, glutamic acid, MnO_2_, Vitamin C, KMnO_4_, and blank. The fluorescence of solutions was measured after the reaction.

### Fluorescence imaging of GGT activity in living cells

U87MG human glioma cells and L02 normal human hepatocytes were cultured in the standard cell culture medium containing 10% fetal bovine serum and 1% penicillin/streptomycin, respectively.

U87MG cells were seeded in a glass-bottom dish and allowed to grow for 24 h. Then NRh-G-NPs (5.8 μg/mL) were added, and the dish was incubated at 37 °C for a different time (0-20 min). To inhibit GGT activity, cells were respectively pretreated with azaserine (0.5 mM and 1.0 mM) for 1 h 35. Then NRh-G-NPs (5.8 μg/mL) were added, and the cells were incubated at 37 °C for another 20 min. L02 cells were seeded in a glass-bottom dish and allowed to grow for 24 h. To activate GGT activity, cells were pretreated with sodium hippurate (1.0 mM) for 1 h 36. Then, NRh-G-NPs (5.8 μg/mL) were added, and the cells were incubated at 37 °C for another 20 min. After replacing the medium, the fluorescence images were captured on a confocal fluorescence microscope 37, 38.

### Fluorescence imaging to indicate tumor location *in vivo*

U87MG tumor model was established by subcutaneous injection of U87MG cells in 4-week-old female nude mice. Tumors were allowed to grow to a diameter of 3-6 mm. NRh-G-NPs (2.9 mg/mL, 100.0 μL) were injected through the tail vein. After injection, the whole-body fluorescence images of the mice were collected at different time points until the fluorescence faded away. When the fluorescence was the strongest, the mice were killed, the tumor and other organs were taken out for imaging *in vitro*. The tumor tissue was sliced and stained with DAPI and the fluorescence images were observed under a confocal fluorescence microscope.

### Photothermal properties of the probe in solution

Different concentrations of NRh-G-NPs aqueous solutions (0, 11.5, 25.9, 34.6, 57.6 μg/mL) were irradiated with 730 nm laser at a power density of 1.0 W/cm^2^ for 5 min. Moreover, different concentrations of NRh-G-NPs (0, 11.5, 25.9, 34.6, 57.6 μg/mL) were incubated with GGT (1.0 U/L) at 37 °C for 30 min and then irradiated with 730 nm laser at a power density of 1.0 W/cm^2^ for 5 min. All the temperature of the solutions was recorded every 30 s.

### Photothermal killing effect of the probe on cancer cells

U87MG cells were cultured in 96 well plates in Dulbecco's modified eagle medium (DMEM) media. In the first group of experiments, cells were treated with different concentrations of NRh-G-NPs (0, 11.5, 25.9, 34.6, 57.6 μg/mL) for 8 h. Then one set was irradiated with a 730 nm laser at a power density of 1.0 W/cm^2^ for 5 min, while the other was not. In another group of experiments, cells were divided into three sets. The first set was incubated with saline for 20 min, the second set was incubated with the probe (5.8 μg/mL) for 20 min, and the third group was incubated with azaserine (0.5 mM) for 1 h and then with the probe (5.8 μg/mL) for 20 min. Each set was treated with laser irradiation for 5 min or not. Cells were then washed thoroughly with PBS buffer, and fresh serum-free DMEM media was added. Next, each well plate with attached cells was treated with 10.0 μL of freshly prepared methyl thiazolyl diphenyl-tetrazolium bromide (MTT) solution (5.0 mg/mL) and incubated for 4 h. Then the supernatant was removed carefully leaving the formazan in the plate. This formazan was dissolved in DMSO, and absorbance was measured at 490 nm. Cell viability was determined according to the above methods. U87MG cells were seeded in a glass-bottom dish in DMEM media. The operation of the second group of experiments was repeated, but all of the three dishes were irradiated with laser for 5 min. Living and dead cells were stained with Calcein AM and PI to verify the photothermal killing effect of NRh-G-NPs 39-41.

### PTT treatment *in vivo*

All animal studies were performed under with guidelines of the Animal Care and Use Committee of China Pharmaceutical University. Female BALB/c nude mice (age, 4-6 weeks) were purchased from Qinglongshan Animal Breeding Farm, Jiangning District, Nanjing. U87MG tumors were established by subcutaneous injection of U87MG cells suspended in 150.0 μL saline (4 × 10^6^) into the armpit of each mouse. The tumor volume was calculated as *V=0.5LW^2^*, where *L* and *W* represent the longitudinal and transverse diameters of the tumor, respectively. The treatment of groups are as follows: (1) saline (100.0 μL), (2) NRh-G-NPs solution (2.9 mg/mL, 100.0 μL), (3) saline with 730 nm irradiation, (4) NRh-G-NPs solution (2.9 mg/mL, 100.0 μL) with 730 nm irradiation. Both saline and the probe were injected into mice via the tail vein. The laser treatment was performed on group (3) and group (4) by irradiating the tumor region for 5 min at a power density of 1.0 W/cm^2^. Each mouse was photographed by an infrared thermal imager to record the temperature changes within 5 min. One mouse from each group was euthanized after 24 h to get their tumors. The tumors were performed a hematoxylin-eosin (H&E) staining assay 38. The tumor volume and body weight were determined every other day for 14 days. After 14 days, mice were euthanized for H&E staining of their major organs. In the meantime, the number of mice alive was recorded daily to obtain the survival curve 42-47.

## Results and Discussion

### Properties and fluorescence characteristics of NRh-G-NPs

**Figure 1:**
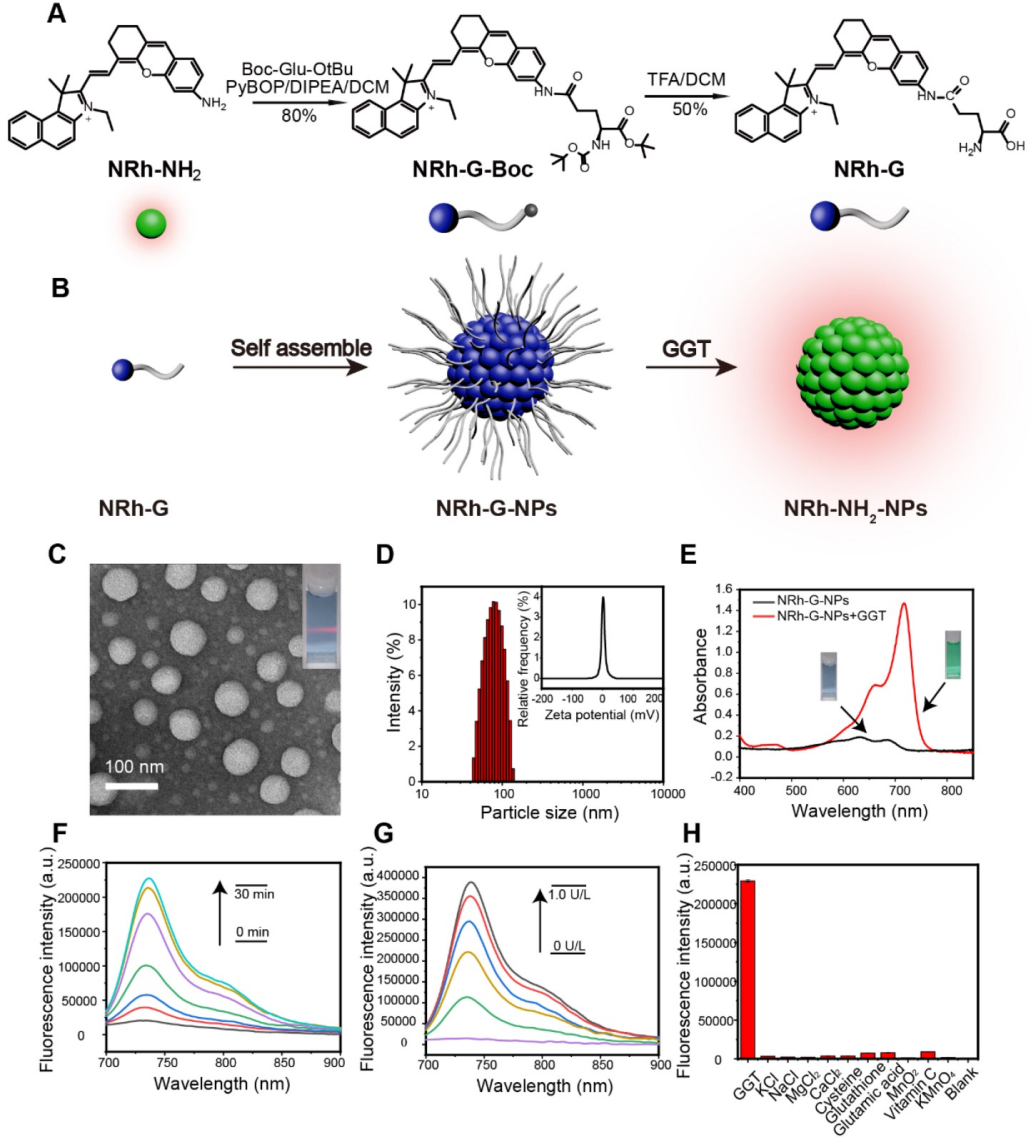
(A) Chemical structure and synthesis steps of NRh-G. (B) Reaction diagram represented by models. NRh-G self-assembled to form NRh-G-NPs, then NRh-G-NPs were cut by GGT to form NRh-NH_2_-NPs and emitted fluorescence. (C) TEM image of nanoparticles and the Tyndall phenomenon of the solution. (D) The hydrodynamic size of the nanoparticles measured by dynamic light scattering (DLS) and the average zeta potential of the dispersion system. (E) UV absorption spectra of the solution before and after the reaction of NRh-G-NPs (5.8 µg/mL) with GGT (0.4 U/L). (F) Fluorescence spectra changes of NRh-G-NPs (5.8 µg/mL) upon addition of GGT (0.4 U/L) for 0-30 min incubation. (G) Fluorescence spectra changes of NRh-G-NPs (5.8 µg/mL) upon addition of different concentrations of GGT (0-1.0 U/L) for 30 min incubation. (H) The fluorescence intensity of NRh-G-NPs (5.8 µg/mL) toward GGT (0.4 U/L), biological fluids (0.5 mM), reducing substances (0.5 mM) and metal ions (0.5 mM). The data are expressed as the mean ± SD (n= 3) (reaction system, PBS buffer, pH 7.4, 37 °C).

The structure of the NRh-G consisted of the GGT-specific substrate γ-Glu and NIR cyanine fluorophore NRh-NH_2_. Amino acid γ-Glu was widely used as a GGT recognition substrate and could be specifically recognized and cut by GGT 48-50. NRh-NH_2_ had strong fluorescence at 740 nm and following reaction with Boc-Glu-OtBu to form intermediate product NRh-G-Boc, the final product NRh-G was generated through Boc deprotection reaction (**Figure 1A**). Due to intramolecular charge transfer (ICT), the absorption of NRh-G was blue-shifted, and the emission of NRh-G was quenched 51. Once NRh-G-NPs were in the high expression environment of GGT, γ-Glu moieties were specifically recognized and cleaved 26, 52, 53. NRh-G-NPs were then transformed into NRh-NH_2_-NPs, showing the NIR absorbance peak at 714 nm and fluorescence recovery at 740 nm (**Figure 1B**).

The structure of NRh-G was examined by ^1^H NMR and HRMS. The peak at m/z 576.28571 (calculated as 575.28568) was ascribed to NRh-G in HRMS (**Figure S5**). After the reaction, the δ 3.76-3.70 (m, 1H) was contributed by the H on the carbon atom connected to the amino group from NRh-G; the δ 8.16 (d, *J* = 8.9 Hz, 1H) and δ 7.82 (d, *J* = 8.9 Hz, 1H) were contributed by the trans-H of alkenyl from NRh-G (**Figure S9**). However, the trans-H of NRh-NH_2_ was at δ 8.77 (d, *J* = 14.4 Hz, 1H) and δ 6.33 (d, *J* = 14.4 Hz, 1H) (**Figure S8**). These data indicated that the targeted probe NRh-G was successfully obtained.

We further evaluated NRh-G-NPs properties in the presence or absence of GGT. The TEM image showed monodispersed NRh-G-NPs with a size around 50 nm (**Figure 1C**). After treatment with GGT, the size of NRh-NH_2_-NPs was decreased to 20-30 nm (**Figure S12**). The hydrodynamic size measured by DLS was decreased from 90 nm (**Figure 1D**) to 70 nm (**Figure S13**) 18. The zeta potential was also changed from -0.3 mV (**Figure 1D**) to 6.0 mV (**Figure S13**). Treatment with GGT for 30 min resulted in the appearance of a UV absorption peak at 714 nm (**Figure 1E**). The fluorescence emission at 740 nm gradually increased as the incubation time (0-30 min, **Figure 1F**) and GGT concentration (0-1.0 U/L, **Figure 1G**) increased 53-57. The reaction carried out in different solutions also displayed superior stability of the probe in a slightly acidic environment (**Figure S14**). The specificity of NRh-G-NPs to GGT was also demonstrated as no fluorescence signal enhancement was observed with other substances (KCl, NaCl, MgCl_2_, CaCl_2_, cysteine, glutathione, glutamic acid, MnO_2_, Vitamin C, KMnO_4_, and blank) (**Figure 1H**) 58, 59.

### Fluorescence imaging in live cells

**Figure 2:**
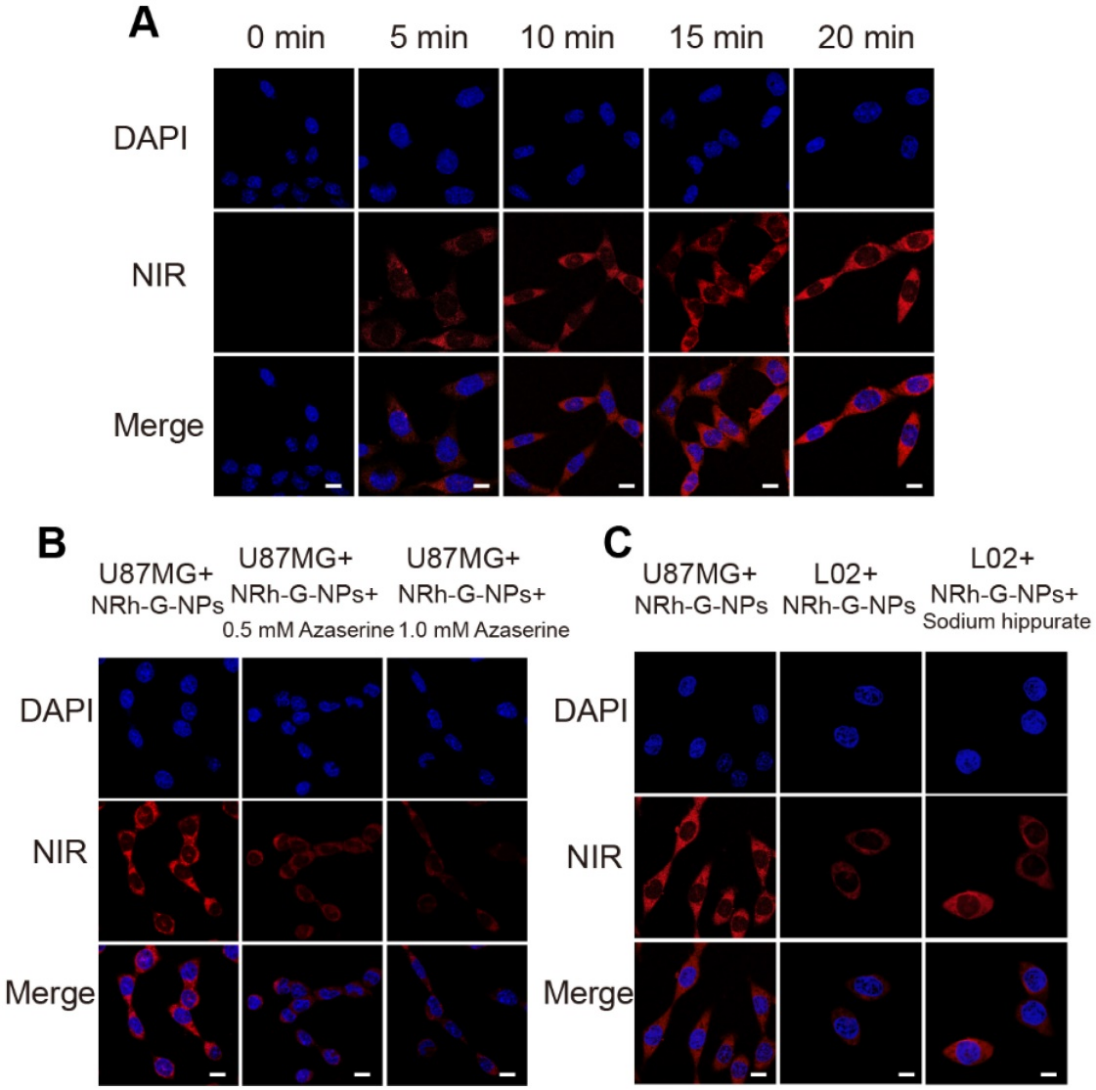
(A) Confocal fluorescence images of U87MG cells incubated with NRh-G-NPs (5.8 µg/mL) for 0, 5, 10, 15, and 20 min. (B) Confocal fluorescence images of U87MG cells incubated with NRh-G-NPs (5.8 µg/mL); confocal fluorescence images of azaserine-pretreated U87MG cells incubated with NRh-G-NPs (5.8 µg/mL). (C) Confocal fluorescence images of U87MG cells and L02 cells incubated with NRh-G-NPs (5.8 µg/mL); confocal fluorescence images of sodium hippurate-pretreated (1.0 mM) L02 cells incubated with NRh-G-NPs (5.8 µg/mL). Scale bar: 10 µm.

The cytotoxicity of NRh-G-NPs was determined by the MTT assay in U87MG cells. The results showed over 90% cell viability with different concentrations of NRh-G-NPs in the range of 0-57.6 μg/mL, indicating the suitability of NRh-G-NPs for live-cell imaging. The activity of GGT in live cells was also detected by NRh-G-NPs. First, U87MG cells incubated with NRh-G-NPs exhibited gradual fluorescence enhancement over time, reaching a plateau value at about 20 min (**Figure 2A**). Next, we pretreated U87MG cells with a specific GGT inhibitor (azaserine) followed by incubation with NRh-G-NPs (5.8 μg/mL) for 20 min 35, 55-57, 60, 61. The fluorescence signal of the cells pretreated with the inhibitor decreased significantly (**Figure 2B**).

Furthermore, human normal liver cells (L02 cells) were used for the comparative test because the content of GGT in U87MG cells was about 5.5-fold higher than in L02 cells (**Figure S15, S16**). When U87MG cells with a high level of enzyme expression were incubated with NRh-G-NPs for 20 min, a strong fluorescence signal was observed. In contrast, L02 cells treated with NRh-G-NPs for the same length of time showed weaker fluorescence 62. When L02 cells were pretreated with an accelerant of GGT (sodium hippurate), then incubated with NRh-G-NPs for 20 min, an enhanced fluorescence signal was observed (**Figure 2C**) 59. Quantitative analysis of the mean fluorescence intensity of the three cell experiments is displayed in **Figure S17-S19**. These results indicated that NRh-G-NPs could distinguish normal cells from cancer cells and were indeed GGT-specific. Since GGT could be an early sensitive marker of oxidative stress, we investigated its synergistic effect with the potential anticancer drug NaBu to induce oxidative stress in cells. As shown in **Figure S20**, a higher NaBu concentration in pretreated cells correlated with stronger fluorescence intensity after incubation with NRh-G-NPs. Simultaneously, the proportion of dead cells increased significantly when pretreated with a high concentration of NaBu. Thus, the anticancer drug NaBu and NRh-G-NPs could be simultaneously used for the diagnosis and treatment of cancer. These results also indicated that NRh-G-NPs had signal stability and could detect enzyme activity at the cellular level.

### Fluorescence imaging *in vivo*

**Figure 3:**
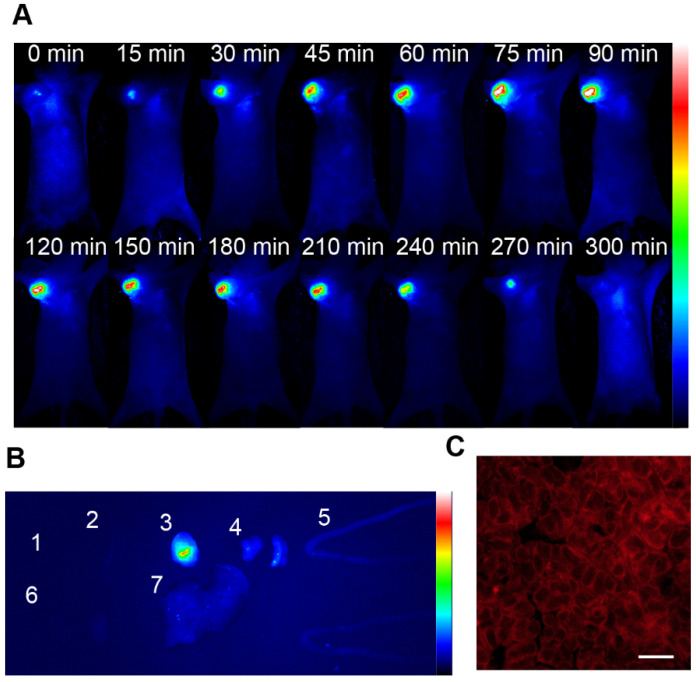
(A) Real-time fluorescence images of mice after NRh-G-NPs (2.9 mg/mL) being injected into mice via the tail vein. (B) Fluorescence images of the heart (1), spleen (2), tumor (3), lung (4), intestine (5), kidney (6), and liver (7) resected from U87MG tumor-bearing mice 1.5 h after tail vein injection of NRh-G-NPs in mice. (C) Confocal z-scan imaging section of a U87MG tumor tissue slice between a depth of 35 µm. Scale bar: 100 µm.

The biocompatibility of NRh-G-NPs was examined using the hemolysis test (**Figure S21**). The nanoparticles were injected into U87MG tumor-bearing nude mice through the tail vein to evaluate the passive targeting ability of NRh-G-NPs to *in vivo* tumors. The whole-body fluorescence was monitored by a bioluminescence imaging system 50, 63, 64. As is evident from **Figure 3A**, the fluorescence gradually increased in the tumors of mice injected with NRh-G-NPs, and the strongest fluorescence was observed in 1.5 h. Subsequently, the fluorescence began to weaken and completely disappeared 5 h after injection. Quantitative analysis showed that the signal-to-noise ratio (SNR) in mice injected with NRh-G-NPs increased significantly within 1.5 h and then began to decrease (**Figure S22**). The mice were dissected 1.5 h after NRh-G-NP injection, and the heart, spleen, tumor, lung, intestine, kidney and liver were excised for biological imaging (**Figure S23**). The fluorescence of tumors was very strong compared with other organs (**Figure 3B**, **Figure S24)**. Confocal images were also taken after slicing tumor tissues (**Figure 3C**). Besides, the three-dimensional reconstruction of the U87MG tumor tissue slice between a depth of 35 μm was performed (**Figure S25**) 50. The results showed that NRh-G-NPs depicted the tumor location by passive targeting and could be used for effective real-time non-invasive imaging of GGT in tumors.

### Photothermal ablation of cancer cells *in vitro*

**Figure 4:**
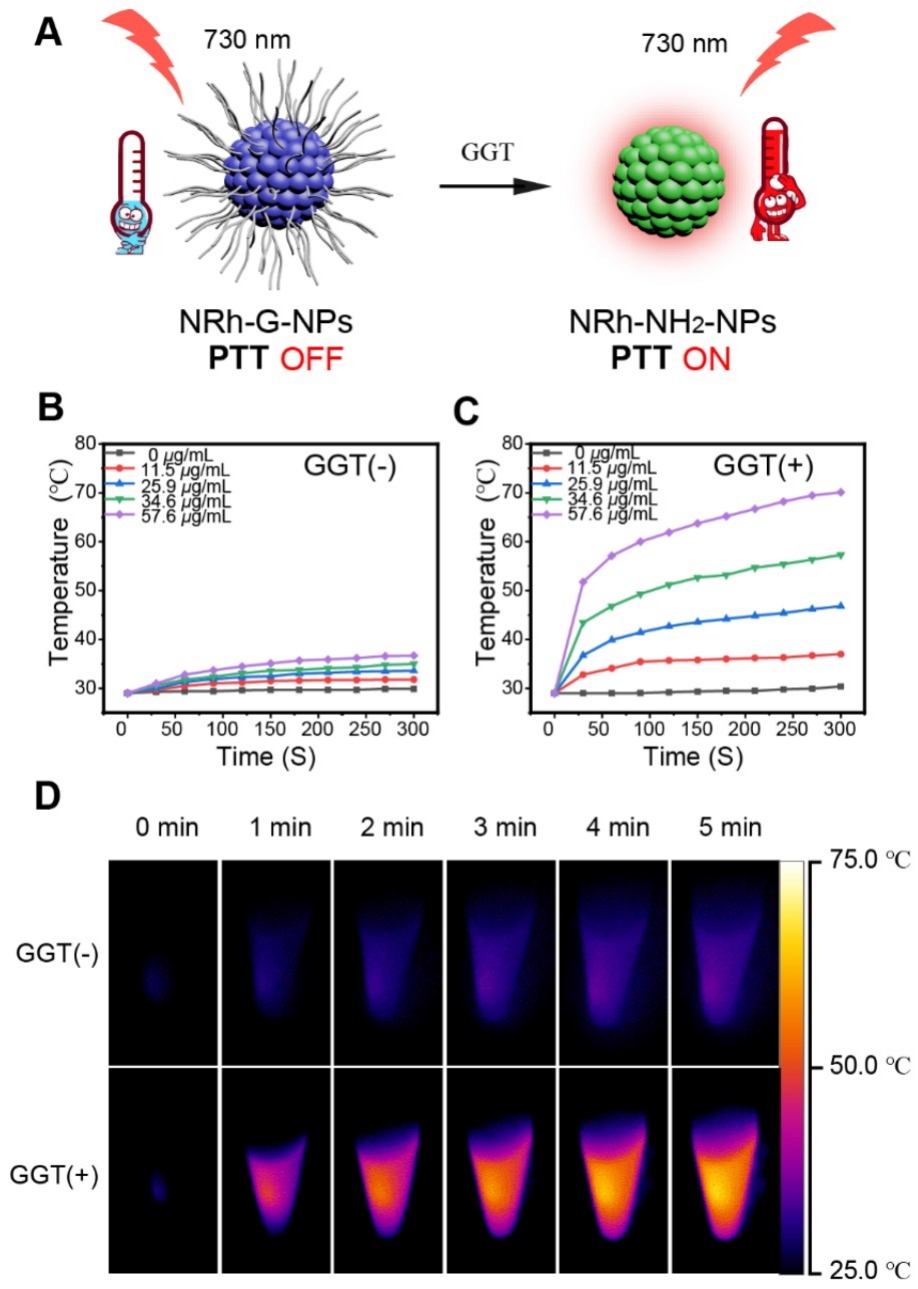
(A) Schematic diagram of the photothermal property turning on. (B) Temperature curves of different concentrations of NRh-G-NPs (0, 11.5, 25.9, 34.6, 57.6 µg/mL) under exposure to the 730 nm light (1.0 W/cm^2^) over a period of 5 min. (C) Temperature curves of different concentrations of NRh-G-NPs (0, 11.5, 25.9, 34.6, 57.6 µg/mL) after reacting with GGT (1.0 U/L) under the exposure to 730 nm light (1.0 W/cm^2^) over a period of 5 min. (D) Infrared thermography of NRh-G-NPs (34.6 µg/mL) without GGT incubation and with GGT incubation.

When GGT specifically cut off the amino acid chains of NRh-G-NPs, the product NRh-NH_2_-NPs showed photothermal effect under 730 nm laser irradiation a photothermal effect under 730 nm laser irradiation (**Figure 4A**). We conducted *in vitro* experiments to demonstrate this phenomenon. Different concentrations of NRh-G-NPs were incubated with or without 1.0 U/L enzyme for 20 min, followed by exposure to 730-nm laser irradiation (1.0 W/cm^2^) for 5min. No significant change in the temperature of different concentrations of NRh-G-NPs without GGT incubation was observed under laser irradiation (**Figure 4B**). However, after enzyme incubation, the speed of the temperature rise and the final temperature reached were proportional to the concentration of NRh-G-NPs under the same laser irradiation condition (**Figure 4C**). Infrared thermographies of NRh-G-NPs (34.6 μg/mL) in **Figures 4B** and **4C,** which were exposed to 730 nm laser for 5 min are displayed in **Figure 4D** 15-18. Moreover, the excellent photothermal stability of the product NRh-NH_2_-NPs was also confirmed (**Figure S26**). We also monitored temperature changes of various concentrations of NRh-NH_2_ under 730 nm laser irradiation for 5 min (**Figure S27, S28**). The results demonstrated that the product of the reaction of NRh-G-NPs with GGT has excellent photothermal property.

**Figure 5:**
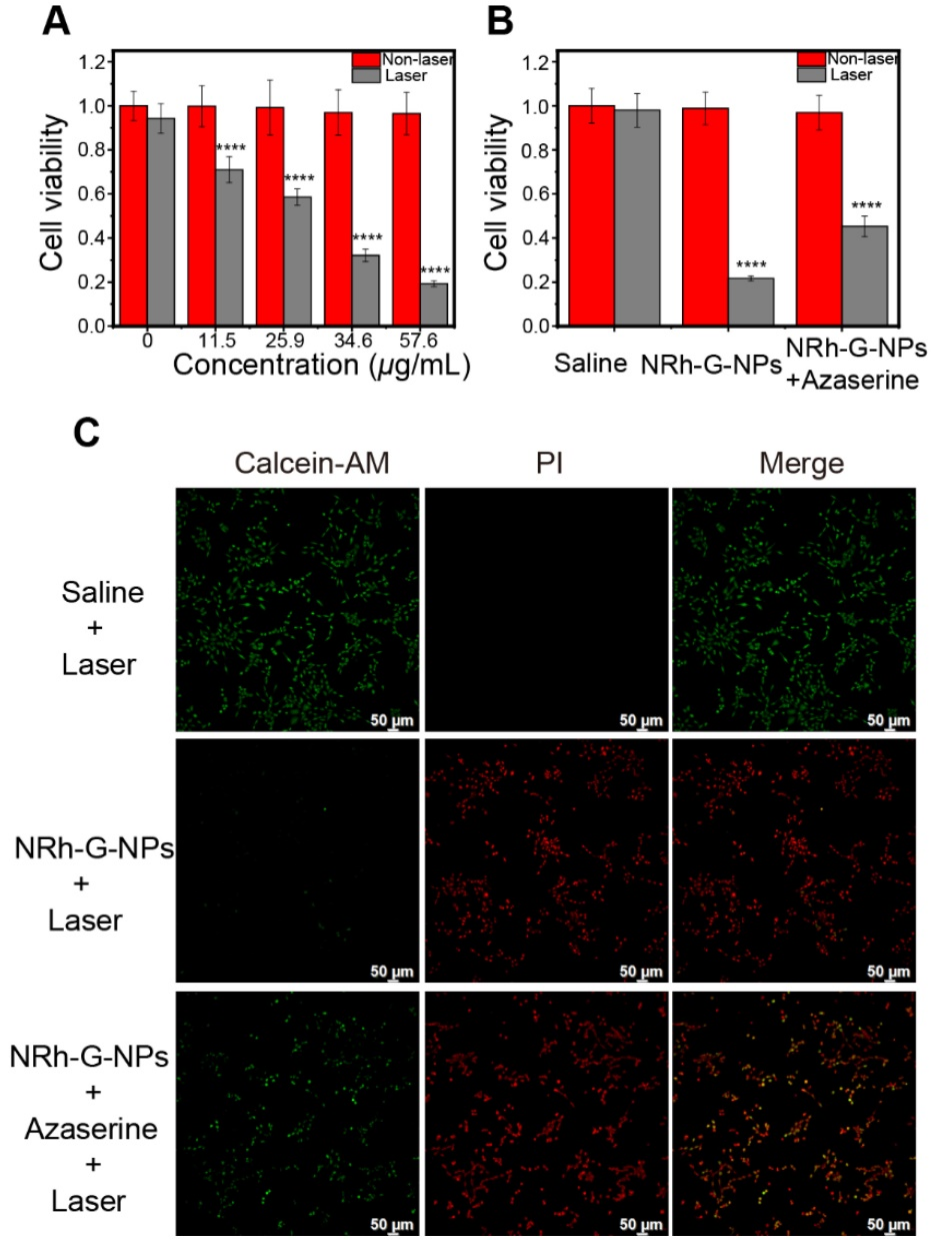
(A) Relative viabilities of U87MG cells before and after different concentrations of NRh-G-NPs induced photothermal therapy under 730 nm laser (1.0 W/cm^2^). (B) Cell viability of U87MG cells with saline or NRh-G-NPs (5.8 µg/mL) or azaserine (0.5 mM) and NRh-G-NPs (5.8 µg/mL) incubation with or without the 730 nm laser (1.0 W/cm^2^). (C) Inverted fluorescence images of Calcein AM/PI stained U87MG cells with saline or NRh-G-NPs (5.8 µg/mL) or azaserine (0.5 mM) and NRh-G-NPs (5.8 µg/mL) incubation before being exposed to 730 nm laser (1.0 W/cm^2^). The data are expressed as the mean ± SD (n= 6). *P < 0.05, **P < 0.01, ***P < 0.001, ****P < 0.0001 compared to the control group.

Based on the standard MTT assay, there was no obvious cytotoxicity of NRh-G-NPs on U87MG cancer cells even at high protein concentrations up to 57.6 μg/mL. Next, we used a 730 nm laser to irradiate the cells incubated with various concentrations of NRh-G-NPs. The cell survival rate decreased significantly with increasing concentrations of NRh-G-NPs (**Figure 5A**). The results also demonstrated the high death rate of cells incubated with NRh-G-NPs after laser irradiation. As expected, the cell death rate was decreased after preincubation with the GGT inhibitor (**Figure 5B, 5C**). Thus, MTT assays and inverted fluorescence imaging of Calcein-AM and propidium iodide (PI) co-stained cells further confirmed the effective and specific photothermal ablation of U87MG cells induced by NRh-G-NPs. These results indicated that NRh-G-NPs could kill tumor cells *in vitro* under specific conditions.

### Photothermal ablation of cancer cells *in vivo*

**Figure 6:**
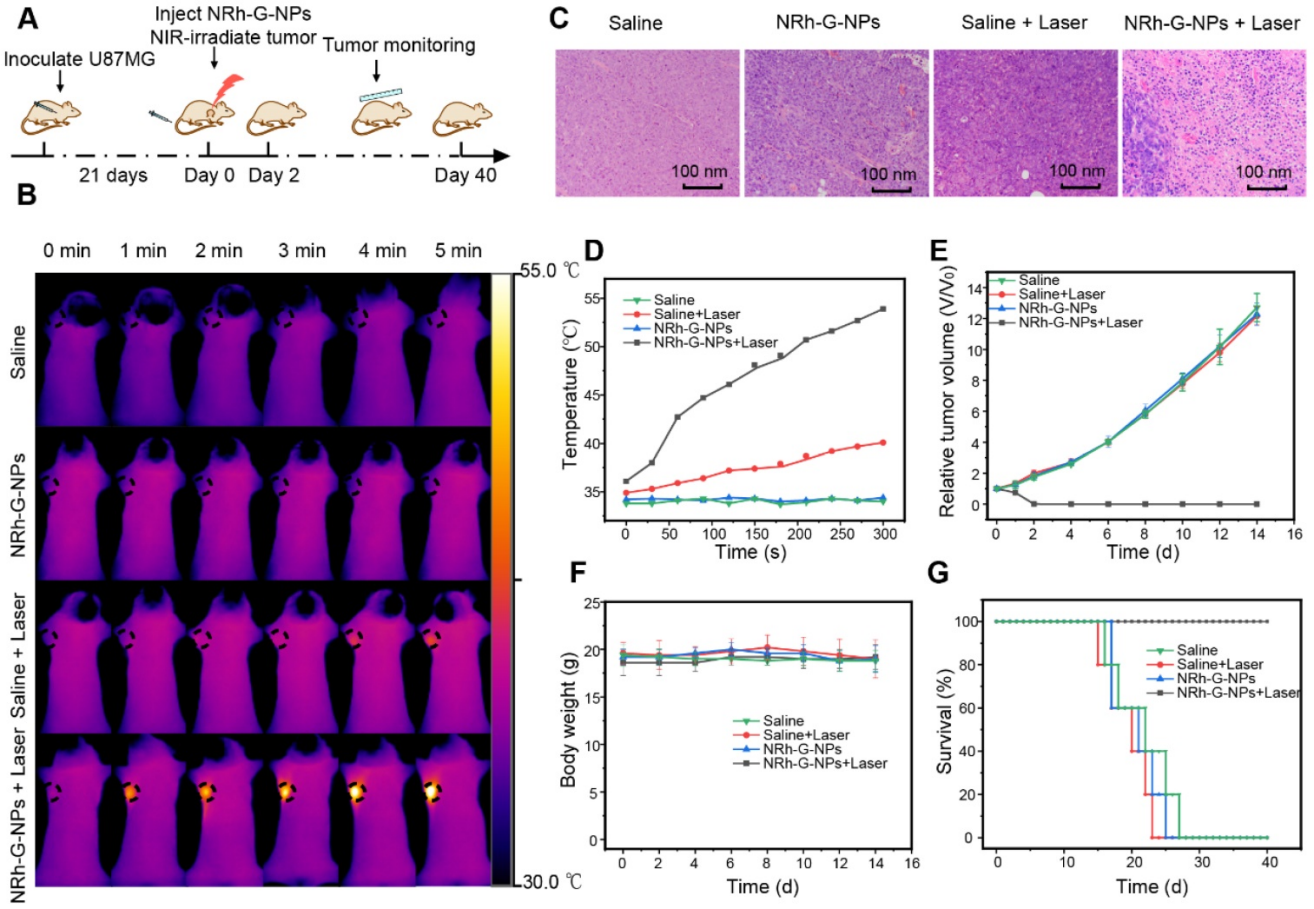
(A) Flow chart of photothermal therapy. (B) Infrared thermography of U87MG tumor-bearing mice with intravenous injection of saline or NRh-G-NPs (2.9 mg/mL) (the first and second row); infrared thermography of U87MG tumor-bearing mice with intravenous injection of saline or NRh-G-NPs (2.9 mg/mL) under the 730 nm laser (1.0 W/cm^2^) irradiation (the third and fourth row). (C) H&E stained tumor slices of the treatment group and the other three control groups. (D) The tumor temperature changes based on the Infrared thermography data in (B). (E) The tumor growth curves of different groups of mice after various treatments. (*P*= 0.0075) (F) The bodyweight of different groups of mice. (G) The survival rate of mice in different treatment groups. The data are expressed as the mean ± SD (n= 5).

Based on the passive targeting of NRh-G-NPs to the tumors *in vivo* and their strong near-infrared absorption, the photothermal therapy *in vivo* was studied on the subcutaneous tumor model of U87MG cells in mice (**Figure 6A**). The body temperature of mice was directly monitored every 1 min by infrared thermography. After intravenous injection of NRh-G-NPs or saline for 1.5 h, the mice were exposed to a 730 nm laser with a power density of 1.0 W/cm^2^ for 5 min and the temperature was monitored by infrared thermography (**Figure 6B**). In mice injected with NRh-G-NPs, the tumor surface temperature increased rapidly from 36 °C to 54 °C under laser irradiation. In contrast, the tumor temperature of other groups of mice did not change significantly under the same irradiation conditions (**Figure 6D**). Histological examination of tumor slices with H&E staining showed that only the tumor structure in the NRh-G-NPs injection group was seriously damaged after laser irradiation (**Figure 6C**). The tumors of mice in the treatment group (NRh-G-NPs + laser) were completely ablated by photothermal therapy over 2 days, and no tumor regeneration was found during the observation period (**Figure S29**). In contrast, the injection of saline or NRh-G-NPs, or only the same power laser irradiation did not affect tumor growth (**Figure 6E**). The photographs of tumors dissected one day after treatment demonstrated that tumors in the “NRh-G-NPs + Laser” group shrank significantly, while the tumors in the other three groups grew in size (**Figure S30**). After 14 days of treatment, mice in all four groups were sacrificed, and the main organs were sectioned and stained with H&E. The results showed that the organs did not suffer any apparent damage (**Figure S31**). There was also no abnormal change in body weight in the four groups of mice (**Figure 6F**). Compared with the average life span of the three control groups, the mice after NRh-G-NP-induced photothermal therapy survived for more than 40 days (**Figure 6G**) 65-67. These results showed that NRh-G-NPs could be used to effectively and precisely treat tumors in animal models without causing damage to other organs.

## Conclusions

We synthesized a γ-glutamyl transpeptidase-activatable near-infrared nanotheranostic which could be applied specifically to detect and treat malignant tumors. The NRh-G-NPs demonstrated no fluorescence or photothermal capability during the blood circulation, but high tumor accumulation due to the EPR effect. Once activated by the enzyme GGT, which is highly expressed in the tumor site, the resulting NRh-NH_2_-NPs showed a strong fluorescent emission that could be used for tumor diagnosis, and showed excellent photothermal conversion properties for photothermal therapy. Both *in vitro* and *in vivo* experiments demonstrated the specificity and effectiveness of NRh-G-NPs for tumor theranostics with no detectable side effects. With excellent biocompatibility, we believe that NRh-G-NPs hold great potential in fluorescence imaging-guided photothermal therapy.

## Supplementary Material

Supplementary methods and figures.

## Figures and Tables

**Scheme 1:**
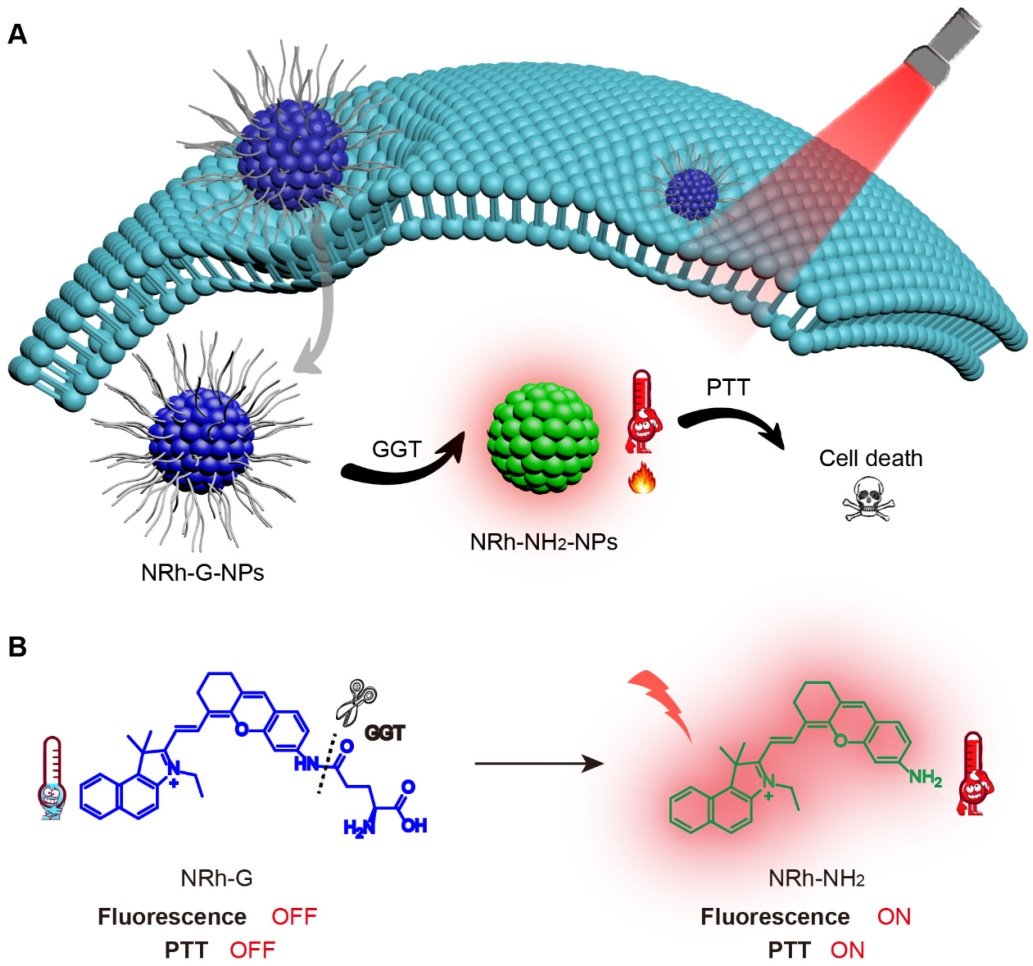
(A) NRh-G-NPs crossed the cell membrane of cancer cells and were specifically cleaved by GGT to form NRh-NH_2_-NPs, which were turned on with fluorescence and photothermal properties. (B) The reaction in which the γ-Glu of NRh-G was specifically cut off by GGT to produce NRh-NH_2_.

## Abbreviations

GGT: γ-glutamyl transpeptidase
γ-Glu: γ-glutamic acid
NIR: near-infrared
PTT: photothermal therapy
MS: mass Spectrum
HRMS: high resolution mass spectrometry
NMR: nuclear magnetic resonance
HPLC: high performance liquid chromatography
TEM: transmission electron microscope
DLS: dynamic light scattering
DMEM: Dulbecco's modified eagle medium
MTT: methyl thiazolyl diphenyl-tetrazolium bromide
H&E: hematoxylin-eosin
analysis of variance): ANOVA
ICT: intramolecular charge transfer
SNR: signal to noise ratio
PI: propidium iodide
