# Supplementary material for: γ-Glutamyl transpeptidase-activatable near-infrared nanoassembly for tumor fluorescence imaging-guided photothermal therapy
Source: Theranostics. 2021 May 13;11(14):7045–56. doi: 10.7150/thno.60586 (PMC8171106; doi:10.7150/thno.60586)
Supplement: Supplementary file 1 — Supplementary methods and figures. [file thnov11p7045s1.pdf]

Supporting information

γ-Glutamyl transpeptidase-activatable near-infrared nanoassembly for tumor fluorescence imaging-guided photothermal therapy

Fangyuan Zhou[a], Shikui Yang[a], Chao Zhao[a], Wangwang Liu[a], Xufeng Yao[a], Hui Yu[a], Xiaolian Sun[b]*, Yi Liu[a]*
[a] School of Engineering, China Pharmaceutical University, Nanjing 211198, P. R. China
[b] State Key Laboratory of Natural Medicines, Key Laboratory of Drug Quality Control and Pharmacovigilance, Department of Pharmaceutical Analysis, China Pharmaceutical University, Nanjing 211198, P. R. China
[*] Corresponding Authors: Xiaolian Sun, Yi Liu
E-mail address: xiaolian_sun@cpu.edu.cn (Xiaolian Sun), yiliu@cpu.edu.cn (Yi Liu)

1. Synthesis.

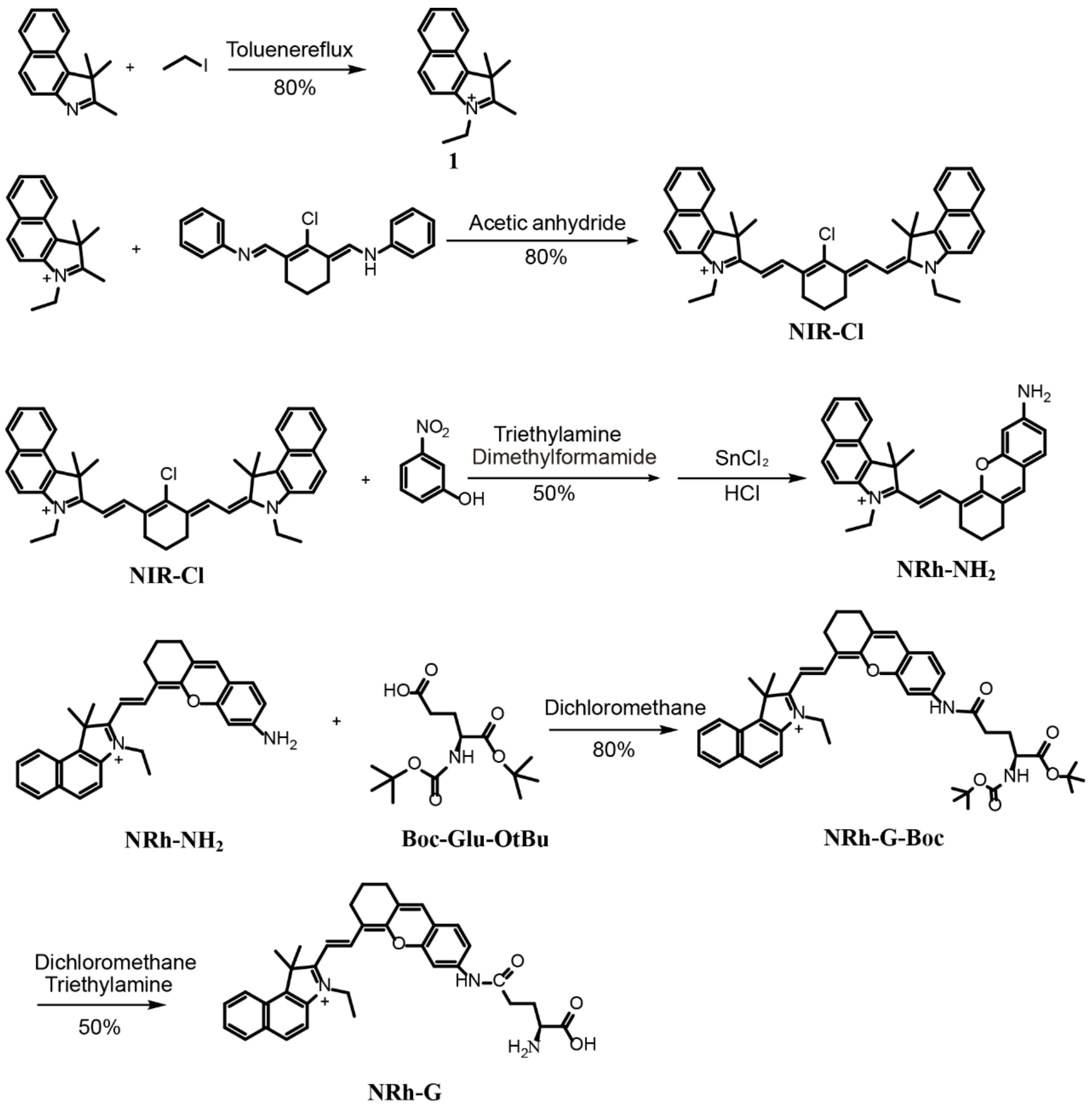

Figure S1. The synthesis process of probe NRh-G.

1.1 Synthesis of compound 1:

1, 1, 2-Trimethylbenz[e]indole (2.09 g， 10.0 mmol) and iodoethane (1.56 g， 10.0 mmol) were placed in a flask containing dry toluene (15.0 mL), and the mixture was stirred at 130 °C with solvent reflux under the nitrogen atmosphere for 8 h. The solvent was removed under vacuum filtration and washed with ether to produce Compound 1 as the blue solid (2.92 g, yield 80%). ^{1}H NMR (400 MHz, MeOD) δ 8.21 (ddd, *J* = 52.0, 39.4, 8.2 Hz, 4H), 7.77 (dd, *J* = 32.2, 7.1 Hz, 2H), 4.86 (s, 3H), 4.72 (d, *J* = 6.9 Hz, 2H), 1.86 (s, 6H), 1.65 (d, *J* = 6.3 Hz, 3H).

1.2 Synthesis of NIR-Cl:

Compound 1 (2.92 g, 8.0 mmol) and N-((2-Chloro-3((phenylimino)methyl)cyclohex-2en-1-ylidene)methyl)aniline hydrochloride (1.44 g, 4.0 mmol) was added into a flask containing dry acetic anhydride and potassium acetate under the nitrogen atmosphere. The mixture was stirred at 60 °C for 3 h. The solvent was hydrolyzed by sodium bicarbonate and removed under vacuum filtration to produce NIR-Cl as the red solid (2.35 g, yield 80%). ^{1}H NMR (400 MHz, MeOD) δ 8.33 (t, *J* = 12.1 Hz, 1H), 8.24 (t, *J* = 12.4 Hz, 1H), 8.17 (d, *J* = 7.6 Hz, 1H), 8.06 (d, *J* = 8.5 Hz, 1H), 7.87-7.77 (m, 1H), 7.73 (d, *J* = 7.0 Hz, 1H), 4.86 (s, 3H), 4.72 (d, *J* = 6.9 Hz, 2H), 1.86 (s, 6H), 1.65 (d, *J* = 6.3 Hz, 3H).

1.3 Synthesis of NRh-NH_2:

NIR-Cl (1.47 g, 2.0 mmol) and 3-Nitrophenol (0.42 g, 3.0 mmol) were placed in a flask containing dry N, N-dimethylformamide and triethylamine. The mixture was stirred at 85 °C for 3 h under the nitrogen atmosphere. $SnCl_2$ (3.80 g, 20.0 mmol) dissolved in concentrated HCl (4.0 mL) was added to the above solution. The mixture was stirred at 85 °C for 8 h under the nitrogen atmosphere. Then the solvent was removed under vacuum filtration at 85 °C. The crude product was purified by silica column chromatography using CH_2Cl_2/CH_3OH (v/v, 50/1) as the eluent to produce the NRh-NH_2 compound as the black solid (0.72 g, yield 50%). ^{1}H NMR (400 MHz, MeOD) δ 8.77 (d, *J* = 14.4 Hz, 1H), 8.29 (d, *J* = 8.6 Hz, 1H), 8.10-8.03 (m, 2H), 7.72-7.66 (m, 2H), 7.55 (t, *J* = 7.4 Hz, 1H), 7.52 (s, 1H), 7.38-7.34 (m, 1H), 6.78 (dd, *J* = 5.6, 1.8 Hz, 2H), 6.33 (d, *J* = 14.4 Hz, 1H), 4.40 (q, *J* = 7.2 Hz, 2H), 2.81-2.74 (m, 4H), 2.09 (s, 6H), 2.00-1.95 (m, 2H), 1.52 (t, *J* = 7.2 Hz, 3H). MS (ESI$^+$): calculated for $C_{31}H_{31}N_2O^+$, 447.2 $[M]^+$; found, 447.2 $[M]^+$.

1.4 Synthesis of NRh-G-Boc:

NRh-NH_2 (0.47 g, 1.0 mmol) was dissolved in dry dichloromethane (2.0 mL). 2-(7-Azabenzotriazol-1-yl)-N, N, N', N'-tetramethyluronium hexafluorophosphate (1.40 g, 5.0 mmol), Boc-Glu-OtBu (3.03 g, 10.0 mmol) and N, N-Diisopropylethylamine (20.0 μL) were dissolved in dry dichloromethane (20.0 mL) at 20 °C for 40 min. Then the former was dripped into the latter. The mixture was stirred at room temperature overnight under the nitrogen atmosphere. After the reaction, an appropriate amount of anhydrous magnesium sulfate was added to the solution to remove the water generated by the reaction. After filtration, the solvent

was removed under vacuum filtration at 35 ^{o}C. The crude product was purified by silica column chromatography using CH_2Cl_2/CH_3OH (v/v, 50/1) as the eluent to produce the NRh-G-Boc compound as the dark blue solid (0.38 g, yield 80%). MS (ESI^+): calculated for $C_{45}H_{54}N_3O_6^+$, 732.4 $[M]^+$; found, 732.5 $[M]^+$.

1.5 Synthesis of NRh-G:

NRh-G-Boc (0.73 g, 1.0 mmol), trifluoroacetic acid (20.0 μL) were placed in a flask containing dry dichloromethane (2.0 mL). The mixture was stirred at room temperature overnight. The solvent was removed under vacuum filtration at 35 ^{o}C. The crude product was purified by reversed phase C18 column chromatography using CH_3OH /H_2O (v/v, 4/1) as the eluent to produce the NRh-G compound as a dark blue solid (0.36 g, yield 50%). ^{1}H NMR (400 MHz, MeOD) δ 8.85 (t, *J* = 12.0 Hz, 1H), 8.36 (t, *J* = 9.5 Hz, 1H), 8.16 (d, *J* = 8.9 Hz, 1H), 8.09 (d, *J* = 1.8 Hz, 2H), 7.82 (d, *J* = 8.9 Hz, 1H), 7.78-7.72 (m, 1H), 7.66-7.60 (m, 1H), 7.44 (t, *J* = 9.1 Hz, 1H), 7.37-7.31 (m, 2H), 6.60 (dd, *J* = 16.0, 8.2 Hz, 1H), 4.60-4.51 (m, 2H), 3.76-3.70 (m, 1H), 2.82-2.79 (m, 2H), 2.74 (dd, *J* = 13.3, 6.7 Hz, 4H), 2.30-2.25 (m, 2H), 2.10 (s, 6H), 2.00-1.94 (m, 2H), 1.57 (d, *J* = 7.3 Hz, 3H). ^{13}C NMR (75 MHz, DMSO) δ 175.46, 162.38, 155.91, 154.00, 141.92, 139.67, 139.14, 137.80, 134.36, 132.15, 131.09, 130.55, 129.71, 129.03, 128.38, 128.11, 127.70, 125.71, 123.35, 122.55, 114.22, 113.34, 112.23, 100.43, 96.36, 55.42, 51.46, 46.83, 27.85, 27.18, 24.10, 20.67, 13.00. MS (ESI^+): calculated for $C_{36}H_{38}N_3O_4^+$, 576.3 $[M]^+$; found, 576.4 $[M]^+$. HRMS (ESI^+): calculated for $C_{36}H_{38}N_3O_4^+$, 576.28568 $[M]^+$; found, 576.28571 $[M]^+$.

2. Characterization.

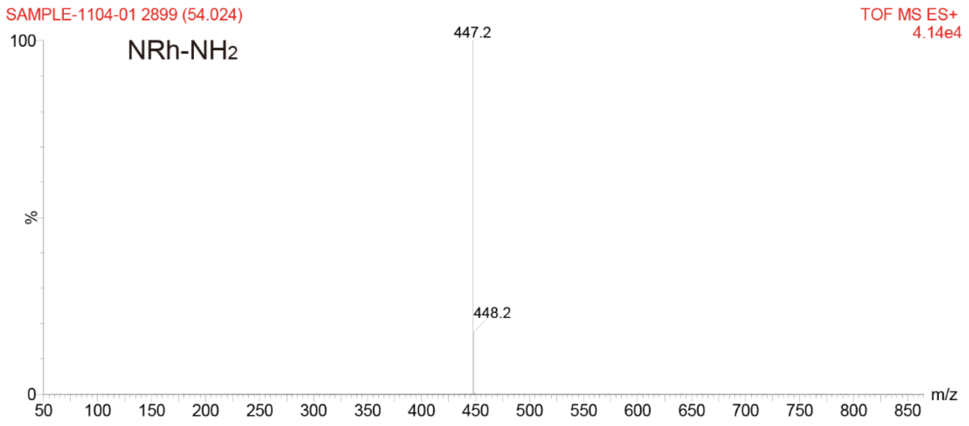

Figure S2. MS of NRh-NH_2.

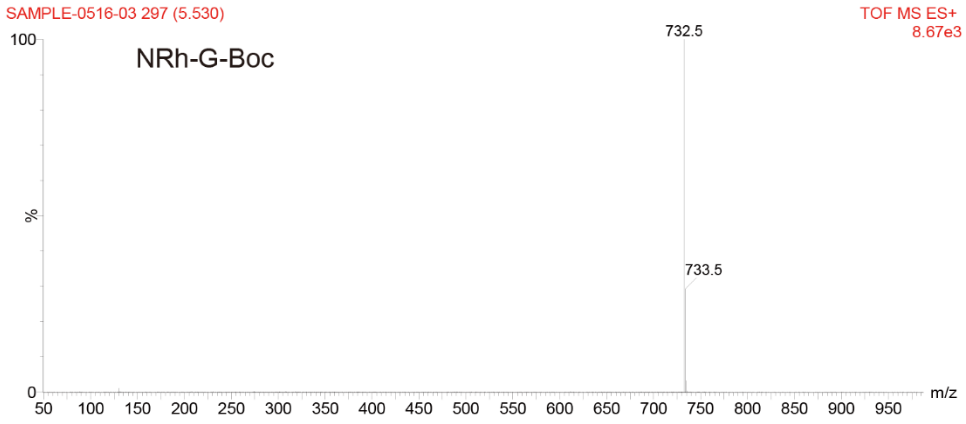

Figure S3. MS of NRh-G-Boc.

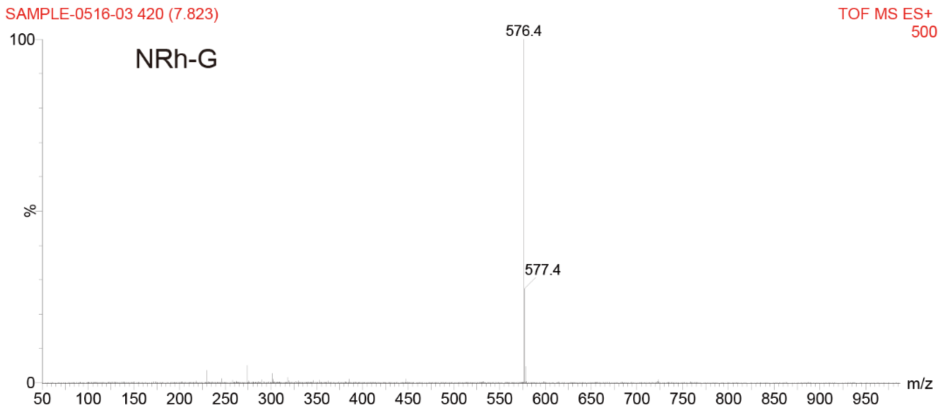

Figure S4. MS of NRh-G.

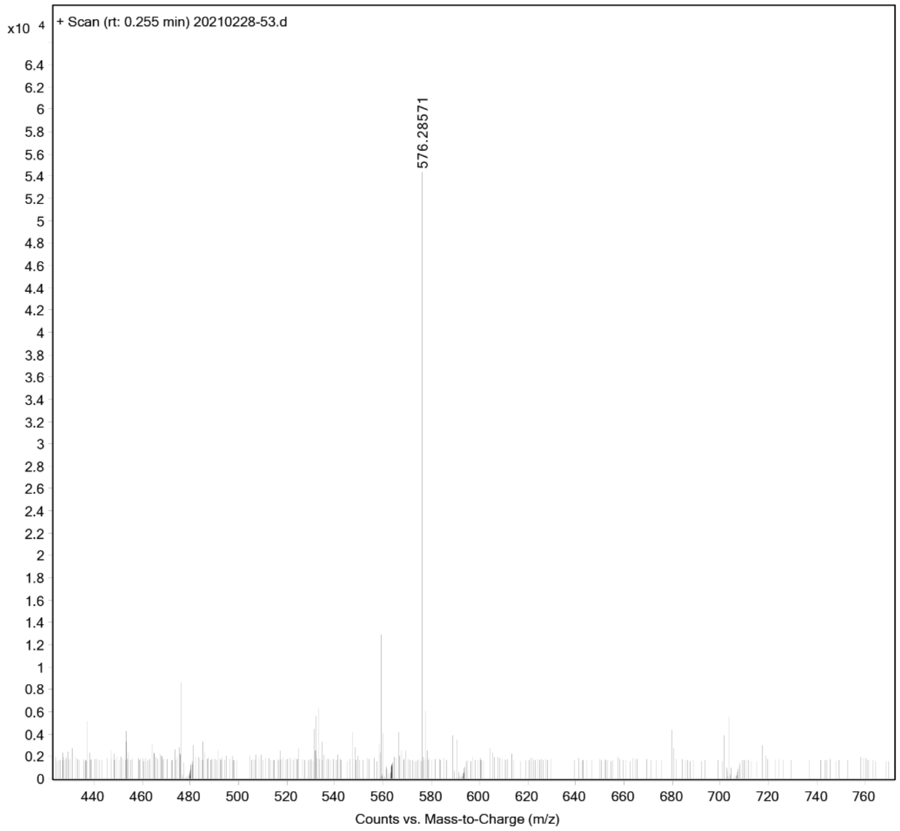

Figure S5. HRMS of NRh-G.

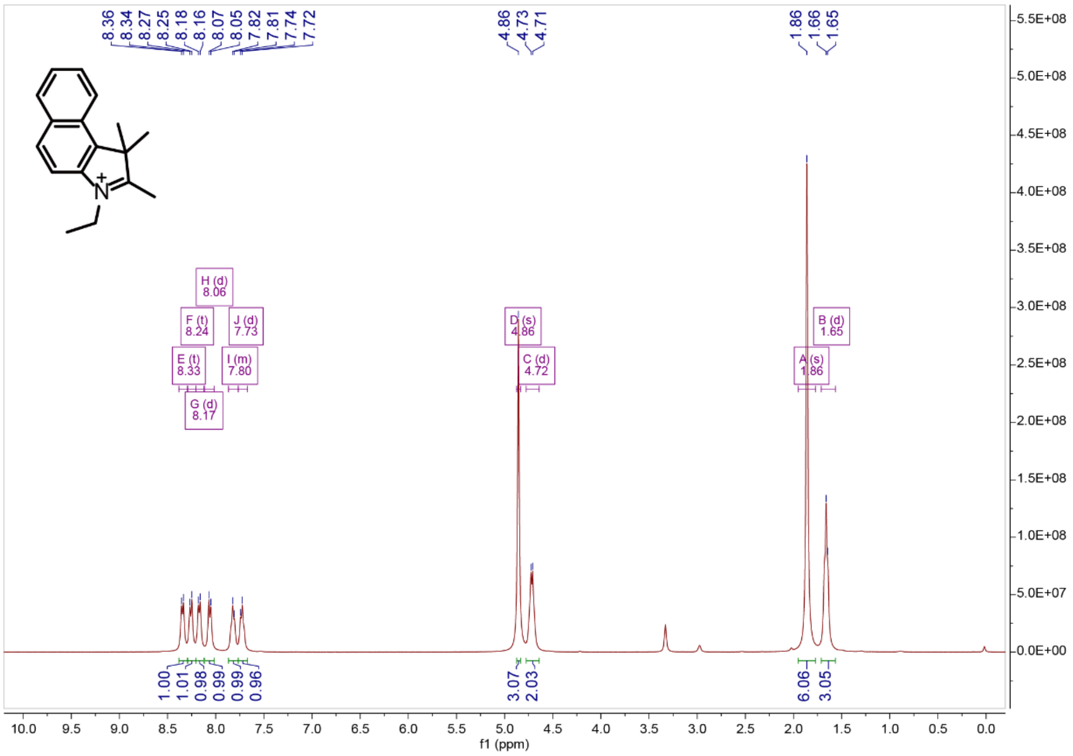

Figure S6. ^{1}H NMR spectra of Compound 1 in MeOD.

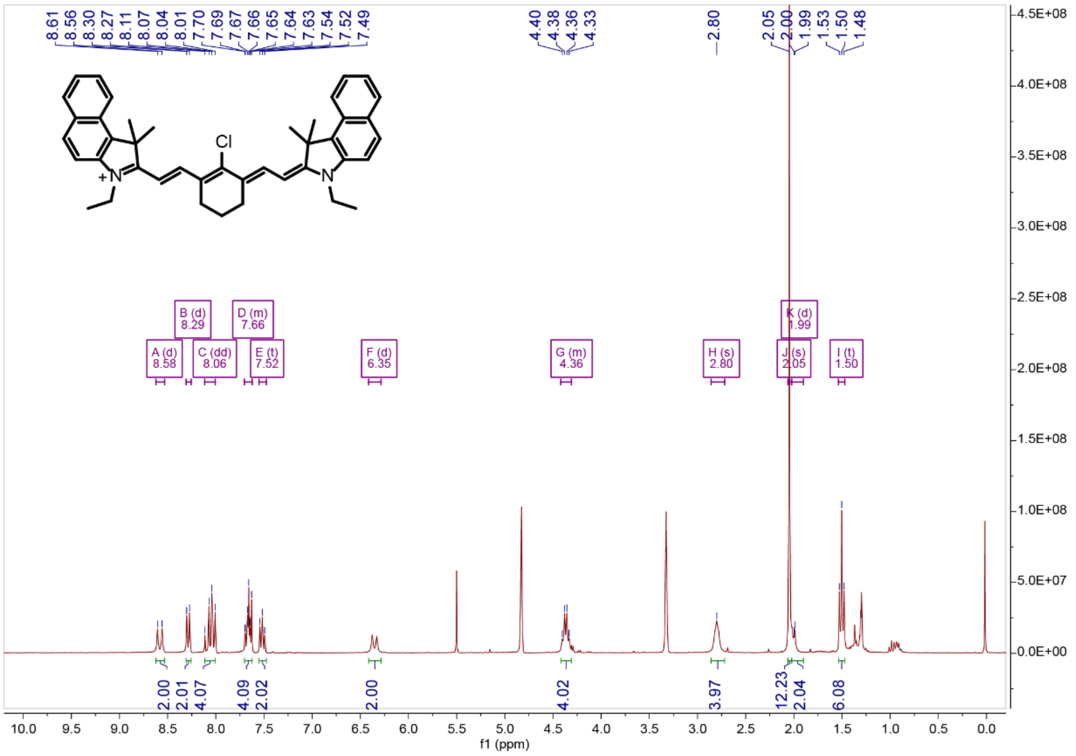

Figure S7. ^{1}H NMR spectra of NIR-Cl in MeOD.

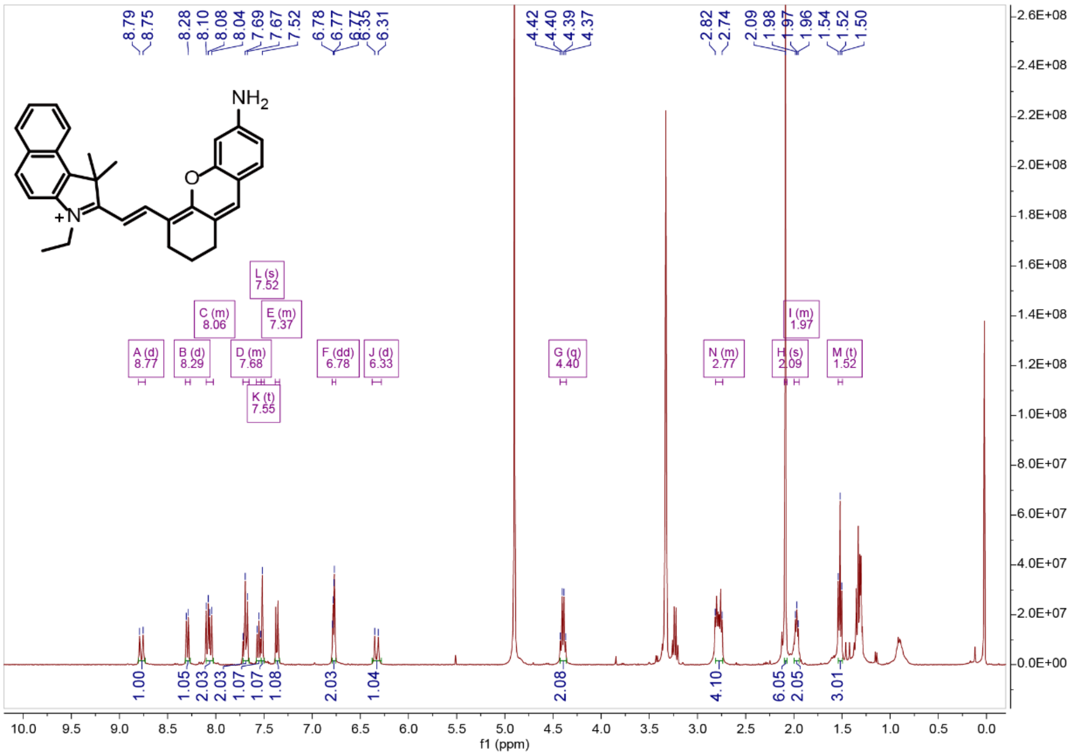

Figure S8. ^{1}H NMR spectra of NRh-NH_2 in MeOD.

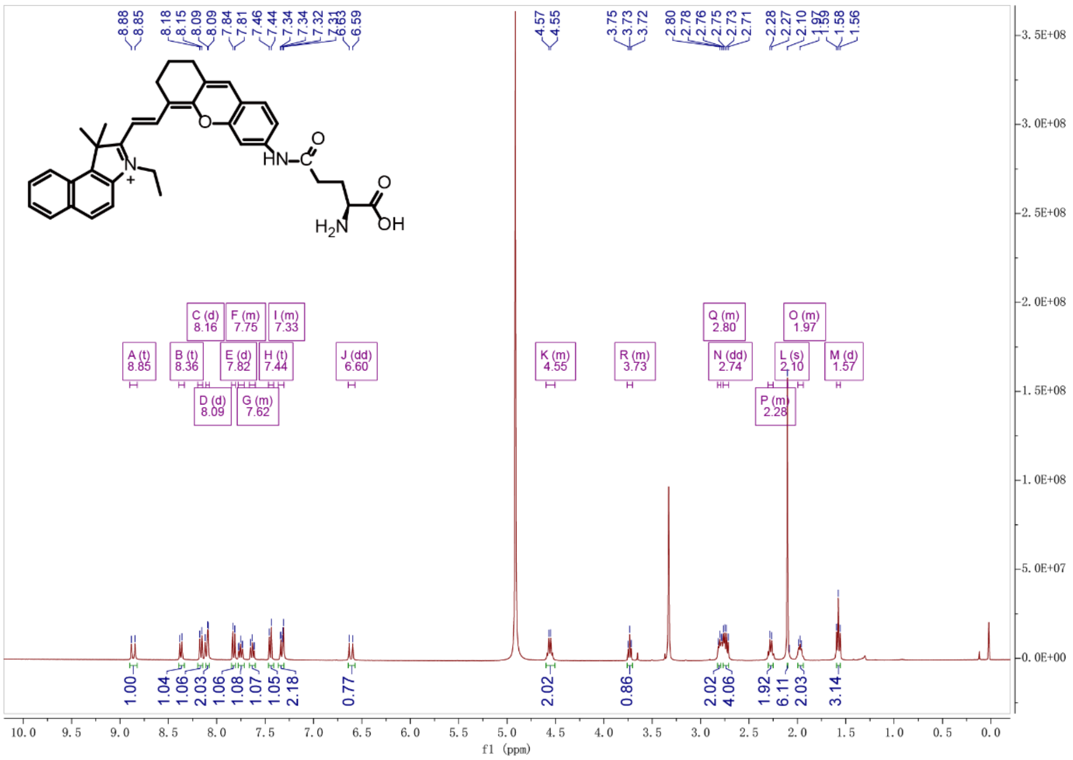

Figure S9. ^{1}H NMR spectra of NRh-G in MeOD.

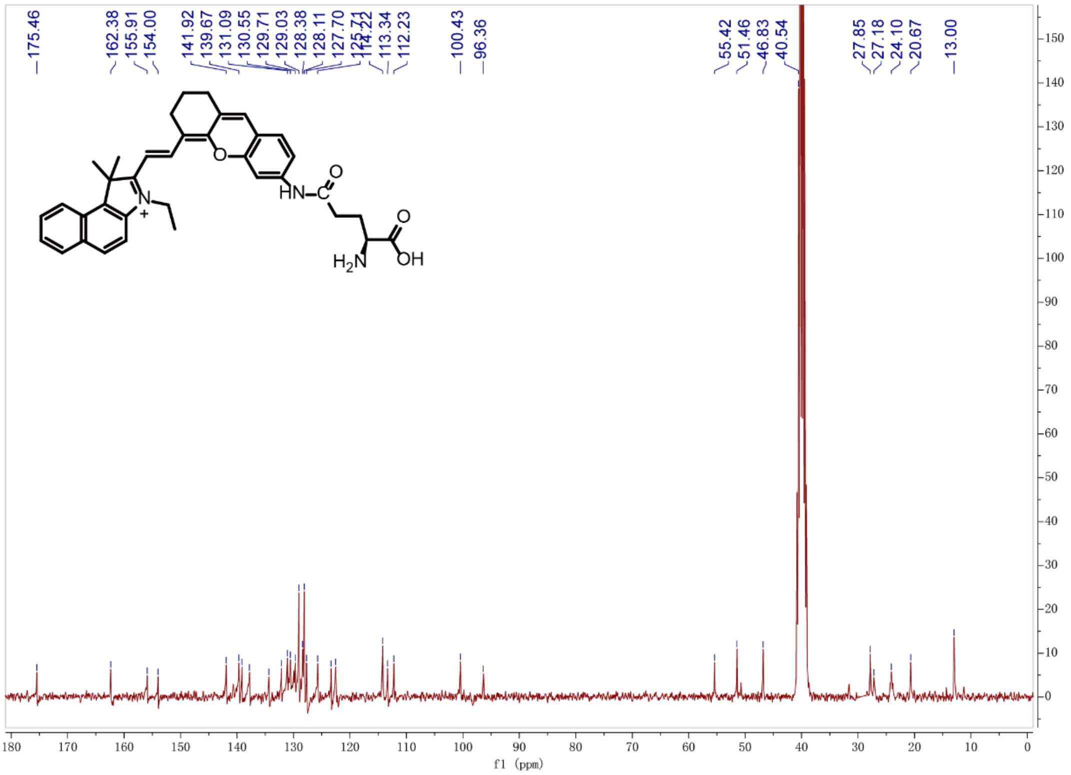

Figure S10. ^{13}C NMR spectra of NRh-G in DMSO.

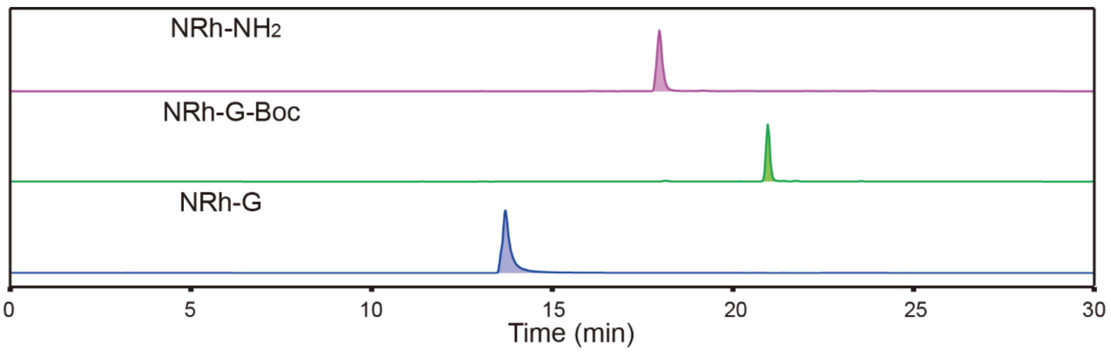

Figure S11. HPLC analyses of $NRh-NH_2$, NRh-G-Boc, and NRh-G.

3. The properties of the solution after NRh-G-NPs react with GGT.

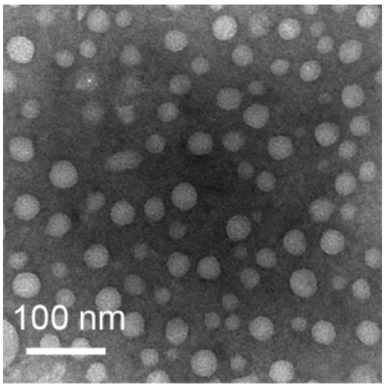

Figure S12. TEM image of the solution after NRh-G-NPs reacts with GGT.

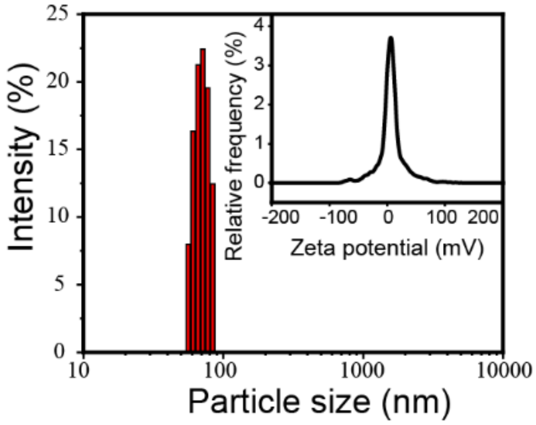

Figure S13. The hydrodynamic size of the nanoparticles measured by DLS and the average zeta potential of the solution after NRh-G-NPs react with GGT.

4. The stability of the NRh-G-NPs in PBS, serum and acid PBS.

PBS with different pH values and serum were selected as solvents. NRh-G-NPs (34.6 μg/mL) and a sufficient amount of GGT were incubated in the above solutions for 30 min, and the fluorescence emission intensity of each solution was measured. It could be seen from the experimental results that NRh-G-NPs had the best stability and the strongest fluorescence emission in neutral PBS. To be compared, the fluorescence of NRh-G-NPs in serum did not decrease badly, indicating that the stability of NRh-G-NPs in serum was also good. As the pH of PBS decreased, the fluorescence emission intensity also decreased, indicating that with the increase of acidity, the stability of NRh-G-NPs will be affected to a certain extent.

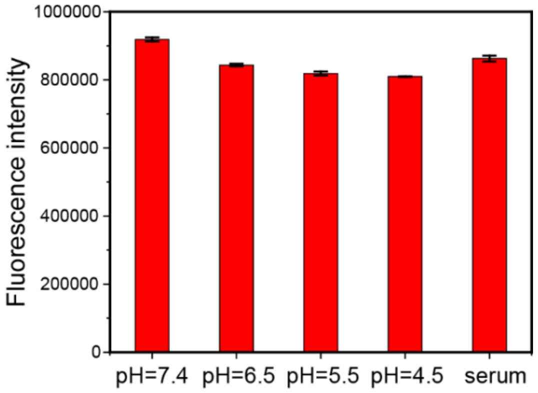

Figure S14. The fluorescent emission stability of the NRh-G-NPs (34.6 μg/mL) in PBS, serum and acid PBS. The data are expressed as the mean ± SD (n= 5).

5. Western blot assay of GGT content in U87MG cells and L02 cells.

According to the western blot assay, it is obvious that the GGT content in U87MG cells is much higher than that in L02 cells. After quantitative analysis of the Western blot result, GGT content in U87MG cells was about 5.5 times that in L02 cells.

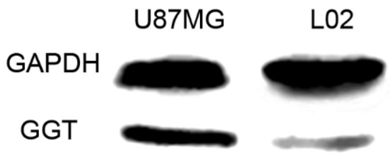

Figure S15. The western blotting of GGT expression in L02 cells and U87MG cells.

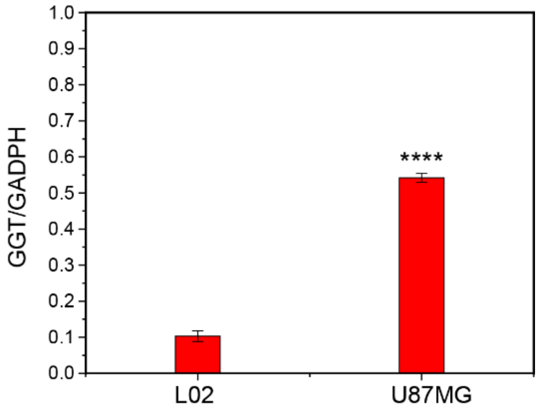

Figure S16. The quantitative analysis of GGT expression in L02 cells and U87MG cells. The data are expressed as the mean ± SD (n= 3). **P* < 0.05, ***P* < 0.01, ****P* < 0.001, *****P* < 0.0001 compared to the L02 group.

6. **Quantitative analysis of fluorescence intensity of cell experiments.**

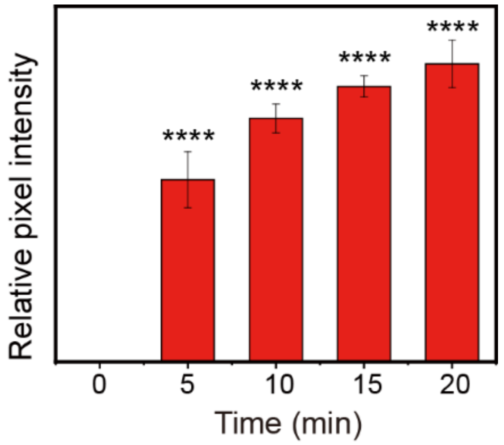

Figure S17. Quantitative analysis of mean fluorescence intensity of cells as indicated in **Figure 3A**. The data are expressed as the mean ± SD (n= 5). **P* < 0.05, ***P* < 0.01, ****P* < 0.001, *****P* < 0.0001 compared to the control group.

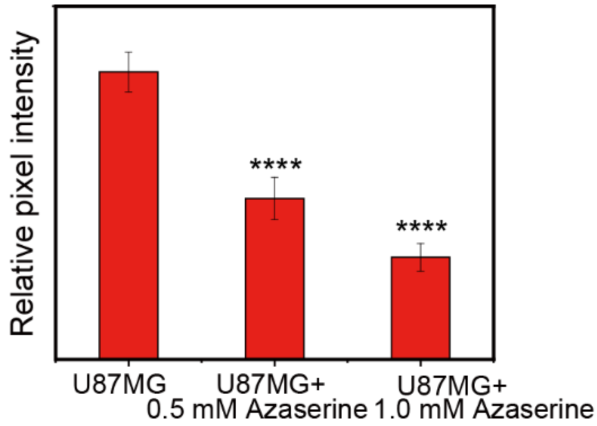

Figure S18. Quantitative analysis of mean fluorescence intensity of cells as indicated in **Figure 3B**. The data are expressed as the mean ± SD (n= 5). **P* < 0.05, ***P* < 0.01, ****P* < 0.001, *****P* < 0.0001 compared to the control group.

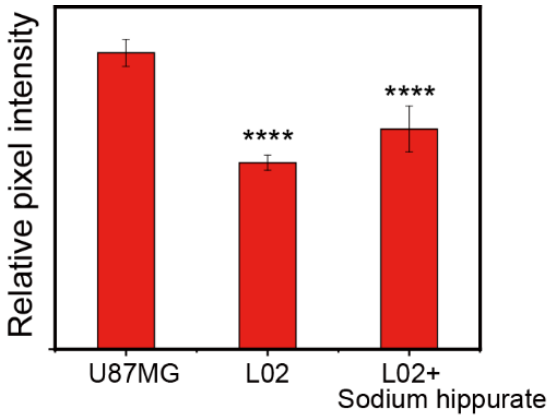

Figure S19. Quantitative analysis of mean fluorescence intensity of cells as indicated in **Figure 3C**. The data are expressed as the mean ± SD (n= 5). **P* < 0.05, ***P* < 0.01, ****P* < 0.001, *****P* < 0.0001 compared to the control group.

7. Synergism effects of NRh-G-NPs and a potential anticancer drug NaBu.

NaBu, as a potential anticancer drug, can cause an imbalance of oxidant/antioxidant ratio by causing the production of reactive oxygen species. And GGT may be an early and sensitive marker for oxidative stress. So oxidative stress occurs in cells pretreated with NaBu, which promotes the expression of GGT. Therefore, treatment with NaBu can promote the high expression of enzymes in cells, enhance the fluorescence after incubation with the probe, thus improve the detection limit. At the same time, it can promote the oxidative stress of cells and accelerate the senescence and apoptosis of cancer cells. For stimulation, the U87MG cells were treated with different concentrations of sodium butyrate (NaBu, 0.5 and 1.0 mM) at 37 °C for 6 h and then incubated with NRh-G-NPs (5.8 μg/mL) for another 30 min. After replacing the medium, the fluorescence images were captured on a confocal fluorescence microscope. At the same time, the living and dead cells were stained to observe the ratio of living and dead cells before and after NaBu treatment (NaBu, 2.0 and 4.0 mM).

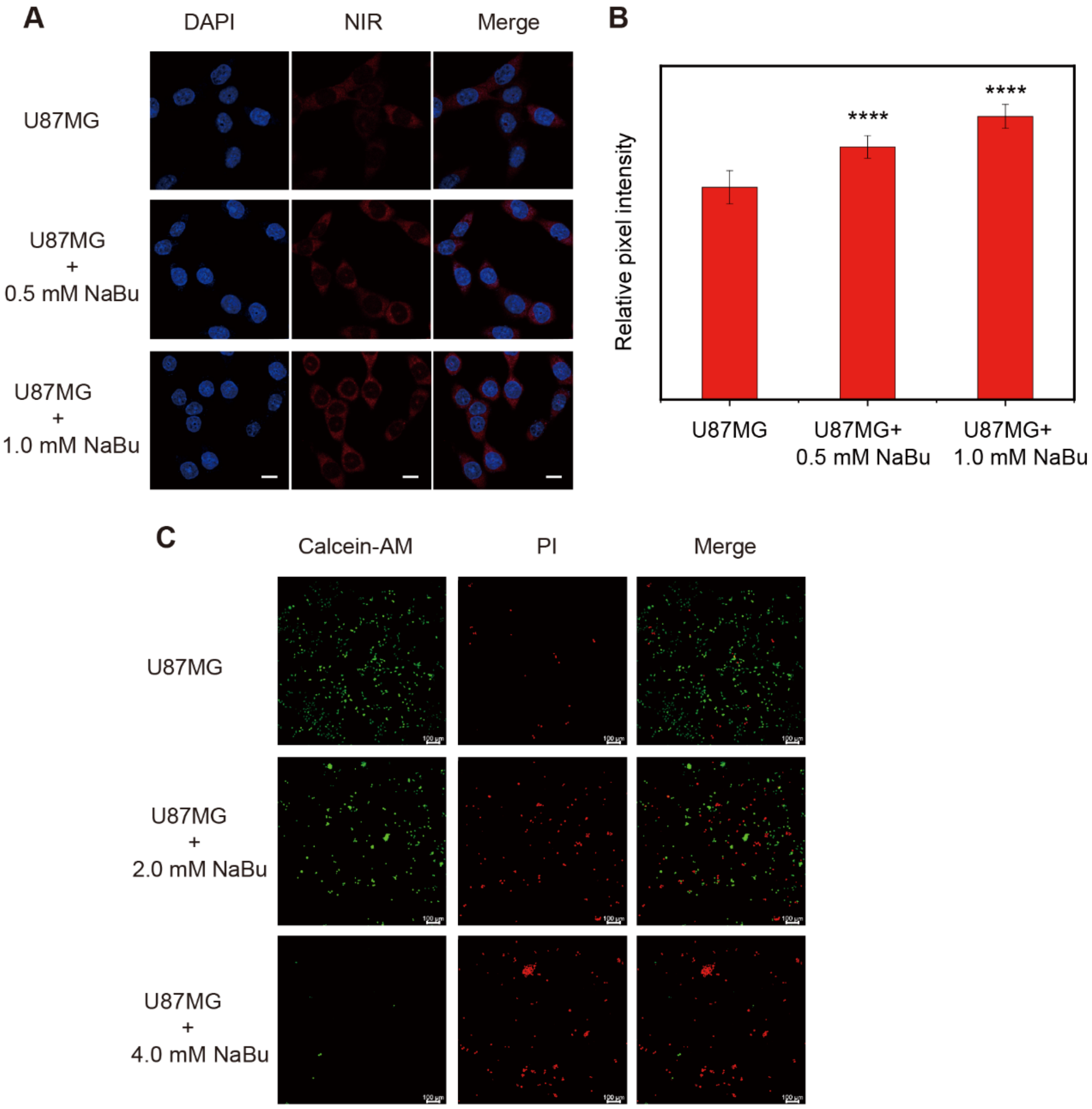

Figure S20. (A) Confocal fluorescence images of U87MG cells and different

concentrations of NaBu-pretreated U87MG cells incubated with NRh-G-NPs (5.8 μg/mL). Scale bar: 10 μm. (B) Fluorescence intensity of U87MG cells and NaBu-pretreated U87MG. (C) Living dead cell staining of NRh-G-NPs-incubated U87MG cells before and after NaBu treatment. The data are expressed as the mean ± SD (n= 5). *P < 0.05, **P < 0.01, ***P < 0.001, ****P < 0.0001 compared to the control group.

8. The hemolysis test of NRh-G-NPs.

Distilled water (800 μL), saline(800 μL) and saline solution of NRh-G-NPs (5.8 μg/mL, 800 μL) were added to the red blood cell suspension (200 μL) of mice respectively, and incubated at 37 °C for 4 h. When the three groups of liquids were observed, it was found that the one group added with water became significantly clear, while the other two groups remained turbid. The absorbance of the supernatant after centrifugation of the three groups was detected, and the hemolysis rate of the probe was obtained to be 1.90%. It can be seen from the above test results that the biocompatibility of the probe is excellent.

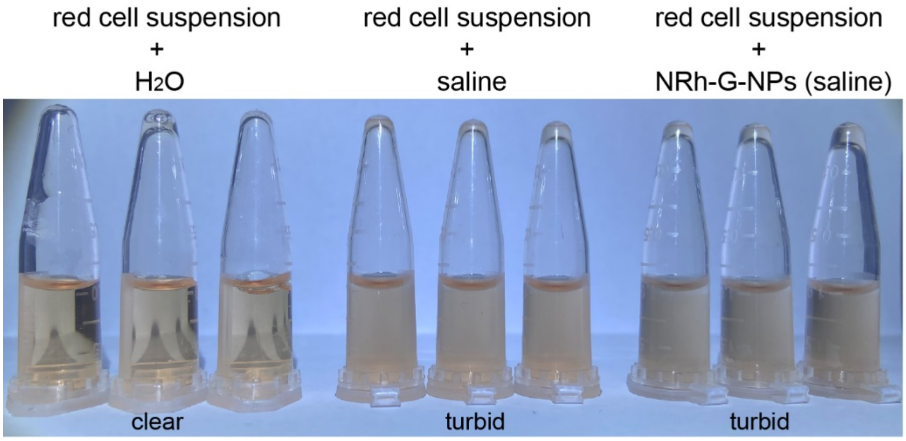

Figure S21. The hemolysis test of NRh-G-NPs (5.8 μg/mL).

9. **Real-time SNR (signal-to-noise ratio) of the tumor region in U87MG tumor-bearing mice.**

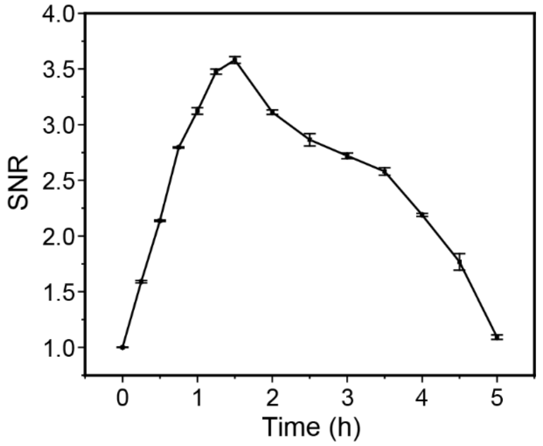

Figure S22. The SNR between the tumor region and the non-tumor region after NRh-G-NPs were injected into U87MG tumor-bearing mice through the tail vein.

10. Bright-field images of organs resected from U87MG tumor-bearing mice.

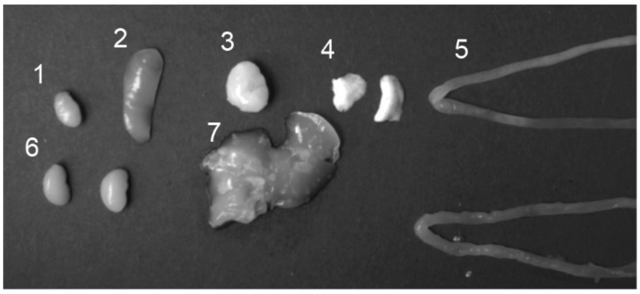

Figure S23. Bright-field images of the heart (1), spleen (2), tumor (3), lung (4), intestine (5), kidney (6), and liver (7) resected from U87MG tumor-bearing mice 1.5 h after tail vein injection of NRh-G-NPs in mice.

11. Fluorescence intensity of different organs in U87MG tumor-bearing mice.

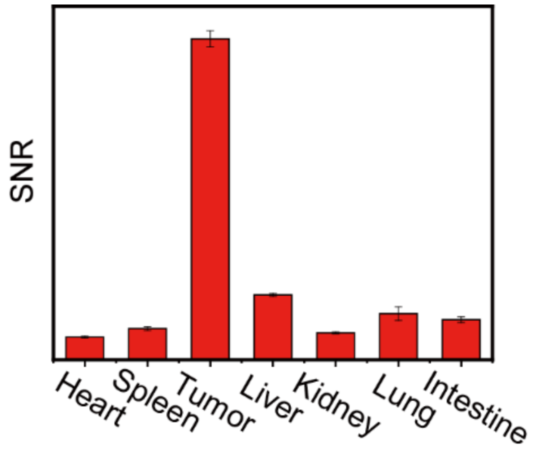

Figure S24. Fluorescence intensity of heart, spleen, tumor, liver, kidney, lung, and intestine resected from U87MG tumor-bearing mice 1.5 h after iv injections of NRh-G-NPs in mice. The data are expressed as the mean ± SD.

12. Three-dimensional reconstruction of a U87MG tumor tissue slice.

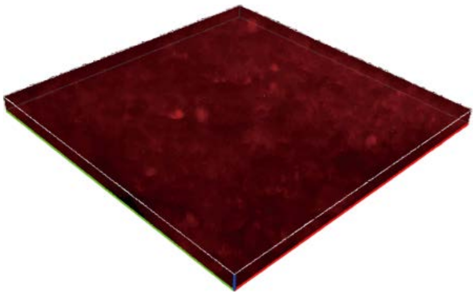

Figure S25. Three-dimensional reconstruction of a U87MG tumor tissue slice between a depth of 35 μm.

13. The photothermal stability and fluorescent emission stability of NRh-NH_2-NPs.

We irradiated NRh-NH_2-NPs (26.8 μg/mL) for 5 min to monitor the temperature rise. After stopping laser irradiation, we continued to monitor the temperature drop, and then conducted laser irradiation after the temperature dropped back to the initial value, and this was repeated four times. We recorded the temperature change of the solution in the whole process. And the fluorescence emission intensity of the solution was measured every time the temperature returned to the initial value. It can be seen that the temperature rise in the four cycles was basically the same, and the fluorescence emission intensity did not significantly decrease. This indicated that the photothermal stability of NRh-NH_2-NPs is quite good.

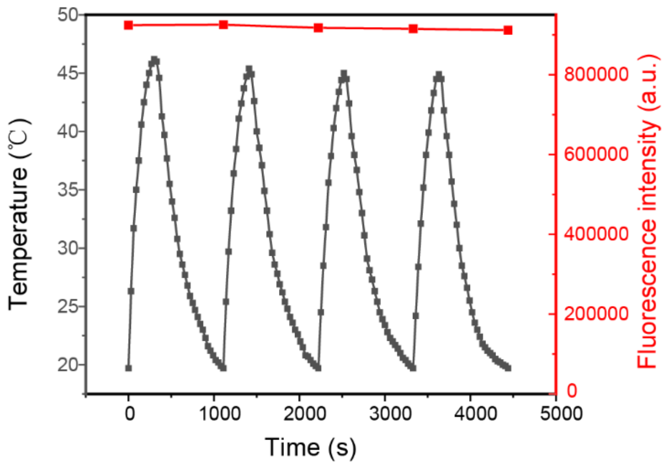

Figure S26. Photothermal stability of NRh-NH_2-NPs (26.8 μg/mL).

14. The photothermal properties of NRh-NH_2.

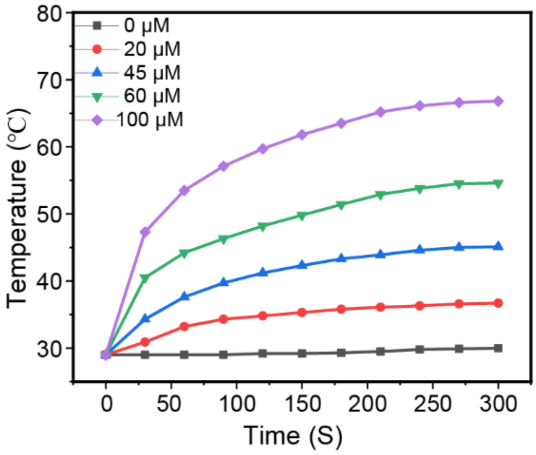

Figure S27. Temperature curves of different concentrations of NRh-NH_2 (0, 20, 45, 60, 100 μM) under exposure to the 730 nm light (1.0 W/cm^2) over a period of 5 min.

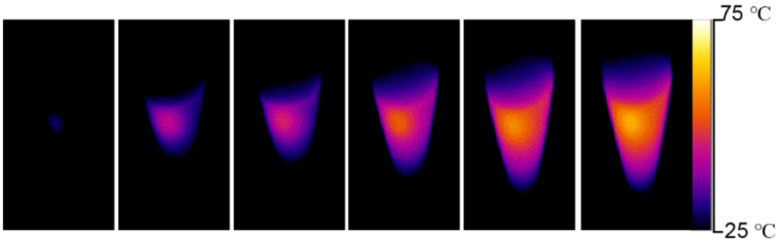

Figure S28. Infrared thermography of NRh-NH_2 (60 μM).

15. Photographs of the one mouse from the treatment group.

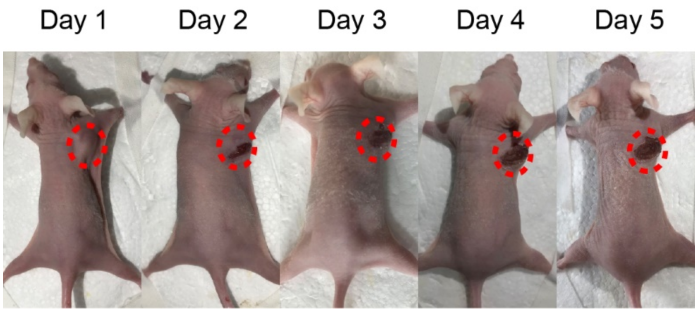

Figure S29. Photographs of the one mouse from the treatment group (NRh-G-NPs + laser) taken daily after treatment. Tumors on mice after NRh-G-NPs injection and laser irradiation were completely eliminated.

16. The photograph of tumors dissected in four groups of mice.

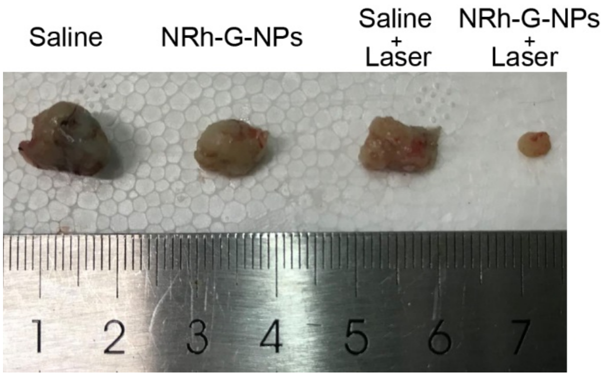

Figure 30. Tumors dissected one day after treatment in four groups of mice.

17. H&E stained organ slices of mice.

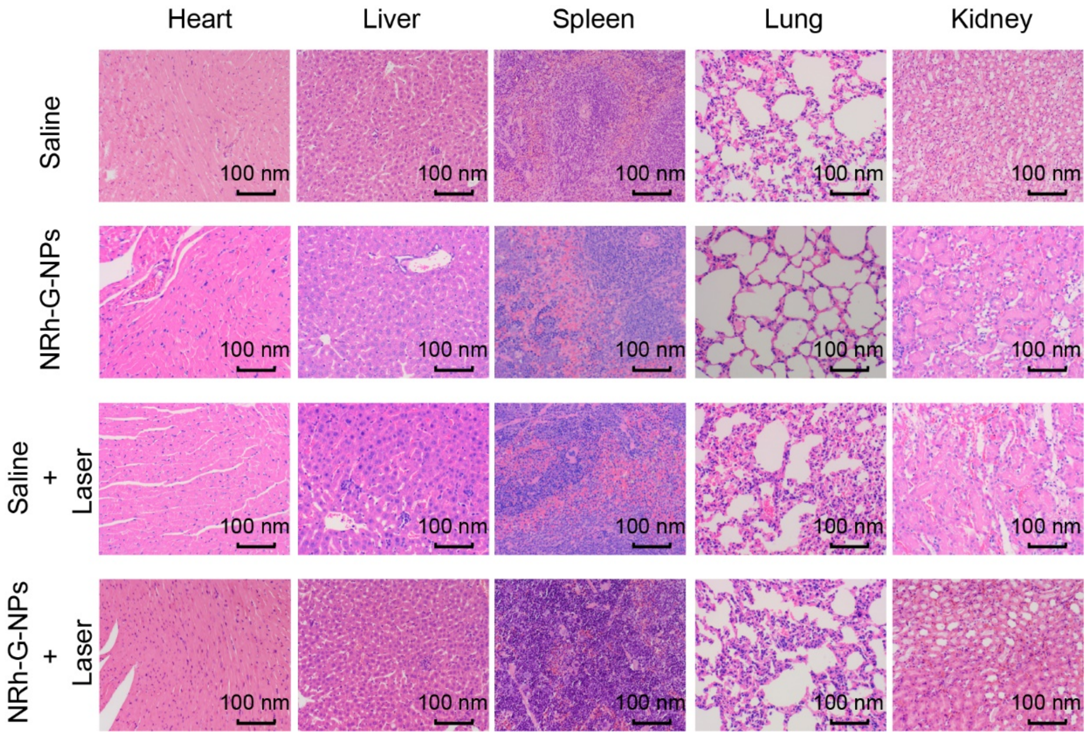

Figure S31. H&E stained organ slices of the treatment group (NRh-G-NPs + laser) and the other three control groups 14 d after treatment.

18. Statistical analysis.

1) Pre-processing of data

In the data collection stage, we must ensure the authenticity and effectiveness of the data sources. And the data are filtered to eliminate the wrong data in advance.

2) Data presentation

Data from all quantitative experiments are presented as “mean ± SD” in the legend.

3) Sample size

The sample size (n) for each statistical analysis is indicated separately in the legend.

4) Statistical methods

statistical test: one-way ANOVA (analysis of variance)

testing level: *P* value

5) Software used for statistical analysis

OriginPro 2018， GraphPad Prism 9
